# Designing Superlubricious Hydrogels from Spontaneous Peroxidation Gradients

**DOI:** 10.1021/acsami.3c04636

**Published:** 2023-08-31

**Authors:** Allison L. Chau, Chelsea E. R. Edwards, Matthew E. Helgeson, Angela A. Pitenis

**Affiliations:** † Materials Department, University of California, Santa Barbara, Santa Barbara, California 93106, United States; ‡ Materials Research Laboratory, University of California, Santa Barbara, Santa Barbara, California 93106, United States; § Department of Chemical Engineering, University of California, Santa Barbara, Santa Barbara, California 93106, United States

**Keywords:** gradient, oxygen inhibition, hydrogel, polyacrylamide, reaction−diffusion model

## Abstract

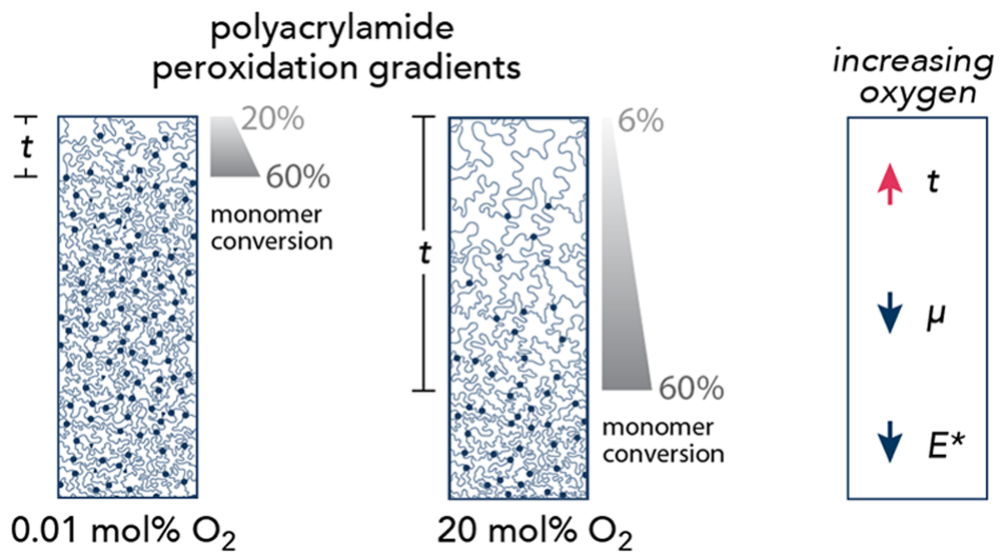

Hydrogels are hydrated three-dimensional networks of hydrophilic polymers that are commonly used in the biomedical industry due to their mechanical and structural tunability, biocompatibility, and similar water content to biological tissues. The surface structure of hydrogels polymerized through free-radical polymerization can be modified by controlling environmental oxygen concentrations, leading to the formation of a polymer concentration gradient. In this work, 17.5 wt % polyacrylamide hydrogels are polymerized in low (0.01 mol % O_2_) and high (20 mol % O_2_) oxygen environments, and their mechanical and tribological properties are characterized through microindentation, nanoindentation, and tribological sliding experiments. Without significantly reducing the elastic modulus of the hydrogel (E* ≈ 200 kPa), we demonstrate an order of magnitude reduction in friction coefficient (from μ = 0.021 ± 0.006 to μ = 0.002 ± 0.001) by adjusting polymerization conditions (e.g., oxygen concentration). A quantitative analytical model based on polyacrylamide chemistry and kinetics was developed to estimate the thickness and structure of the monomer conversion gradient, termed the “surface gel layer”. We find that polymerizing hydrogels at high oxygen concentrations leads to the formation of a preswollen surface gel layer that is approximately five times thicker (*t* ≈ 50 μm) and four times less concentrated (≈ 6% monomer conversion) at the surface prior to swelling compared to low oxygen environments (*t* ≈ 10 μm, ≈ 20% monomer conversion). Our model could be readily modified to predict the preswollen concentration profile of the polyacrylamide gel surface layer for any reaction conditions—monomer and initiator concentration, oxygen concentration, reaction time, and reaction media depth—or used to select conditions that correspond to a certain desired surface gel layer profile.

## 1 Introduction

Low friction, aqueous interfaces are ubiquitous in biology—from articular cartilage protecting synovial joints^1^ to mucin gels coating epithelial surfaces throughout the body (e.g., eyes, ears, respiratory tract, gastrointestinal tract, and reproductive tract).^2−4^ Hydrogels, which are three-dimensional networks of hydrophilic polymers swollen in water, are widely used as synthetic mimics for these biological interfaces due to their high water content (>80%), biocompatibility, tunable mechanical properties, and lubricity.^5−16^ The mechanical and transport properties of cross-linked hydrogel structures are controlled by the average spacing or correlation length between polymer chains, known as the mesh size, ξ. Polymer concentration and cross-link density control mesh size, which can also be tuned after polymerization by solvent quality, pH, and temperature.^17^ Conventionally “homogeneous” hydrogels are assumed to have a characteristic microstructure and mesh size that do not dramatically differ between the surface and the bulk. However, previous studies have demonstrated microscale heterogeneities in homogeneous hydrogels despite exhibiting generally isotropic bulk properties.^18,19^

Investigations of the interplay between chemically cross-linked, homogeneous hydrogel structure and mechanics have led to scaling relationships between the mesh size and elastic modulus in the semidilute regime for good solvents,^20^*E* ∼ ξ^–3^, and friction coefficient,^21^ μ ∼ ξ^–1^. These scaling relationships indicate that increasing ξ reduces *E* and μ concurrently, resulting in a trade-off between strength and lubricity. By contrast, biological hydrogels, such as the extracellular matrix surrounding cartilage, often possess both exceptional load-bearing capabilities and ultralow friction coefficients. For cartilage, these excellent mechanical properties can be attributed to the hierarchical structure and compositional gradient of its extracellular matrix, an entangled network of collagen fibers and proteins.^22,23^

**Figure 1:**
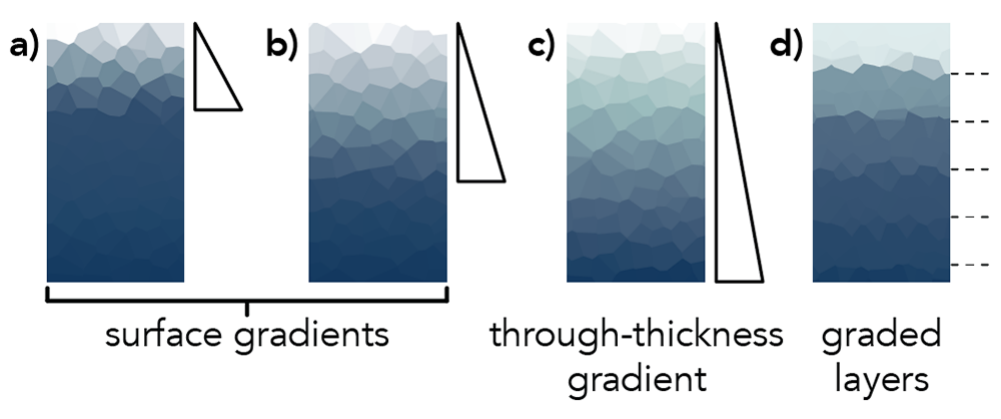
Illustration of hydrogels exhibiting relatively (a) shallow and (b) deep depthwise surface gradients. (c) Depthwise gradient through the entire hydrogel sample thickness. (d) Graded hydrogels with discrete layers of increasing polymer concentration.

To achieve this difficult balance of stiffness and lubricity in synthetic systems, recent investigations have focused on synthesizing and characterizing hydrogels with spatial gradients, where there is a gradual transition in mesh size or cross-linking density with increasing depth (*z*) (Figure 1a–c) as opposed to discrete layers with abrupt, stepwise changes in structure (Figure 1d).^24−27^ There are many established methods used to synthesize hydrogels with spatial gradients, including dip coating,^28^ microfluidics,^25,29^ fluid mixing,^30,31^ controlling substrate thickness,^32^ UV polymerization with photomasks,^24,33−35^ and electrochemical gradients, although these are often time-consuming and low throughput due to their experimental complexity.^36,37^

The simplest method to create hydrogels with polymer concentration gradients at the surface (top 100s of μm) does not require complex synthetic techniques. In 2001, Gong et al. discovered that hydrogels cast on hydrophobic substrates polymerized heterogeneously, forming gels with gradients at the gel–substrate interface.^38,39^ This “substrate effect” was attributed to high interfacial tension between the polymerizing solution and hydrophobic substrate, leading to a polymer concentration gradient that increased from the gel interface to the gel bulk.^40^ Spencer et al. synthesized polyacrylamide hydrogels on substrates with varying surface energies and demonstrated that μ decreased with increasing substrate hydrophobicity; hydrogels cast on glass exhibited higher μ and *E* compared to those cast on polystyrene.^41^ Recently, Simič and Spencer convincingly argued that oxygen permeability of the molding surface is responsible for this “mold effect” rather than surface energy.^42^ This reasoning followed observations that oxygen inhibits free-radical polymerization and affects the interface exposed to air.^42,43^

Oxygen inhibition of free-radical polymerization reactions is a well-known phenomenon in polymer synthesis, particularly in thin films. Dissolved oxygen acts as a radical scavenger, terminating propagation of the polymerizing chain by reacting with polymerizing alky radicals to form stable, chain-terminal peroxyl radicals.^44−54^ This inhibition significantly impedes polymerization where oxygen is abundant and leads to the formation of a liquid surface layer that never gels. There have been several studies examining the kinetics of this phenomenon in photoinitiated acrylate polymer thin film systems^44,46−48,50,52−57^ to estimate the thickness of the unpolymerized layer, with estimates ranging between 1 and 50 μm, depending on the photoinitiator concentration, initiation rate, oxygen concentration, and initial film thickness (Supporting Information (SI), Table S1).^47,49,51,54,55,58^ In general, decreasing ambient oxygen concentration diminishes peroxyl formation and increases double-bond conversion, reducing the thickness of the unpolymerized liquid layer.^44,47,54,55^ Additionally, shortening the curing time by increasing the rate of radical initiation and propagation reduces the effects of oxygen inhibition by minimizing the diffusion time of oxygen.^44,47,54,55^ However, these models did not investigate the structure or thickness of the gel surface that formed below this liquid layer due to oxygen diffusion within the film, despite clear demonstrations of a concentration gradient.^55^

**Figure 2:**
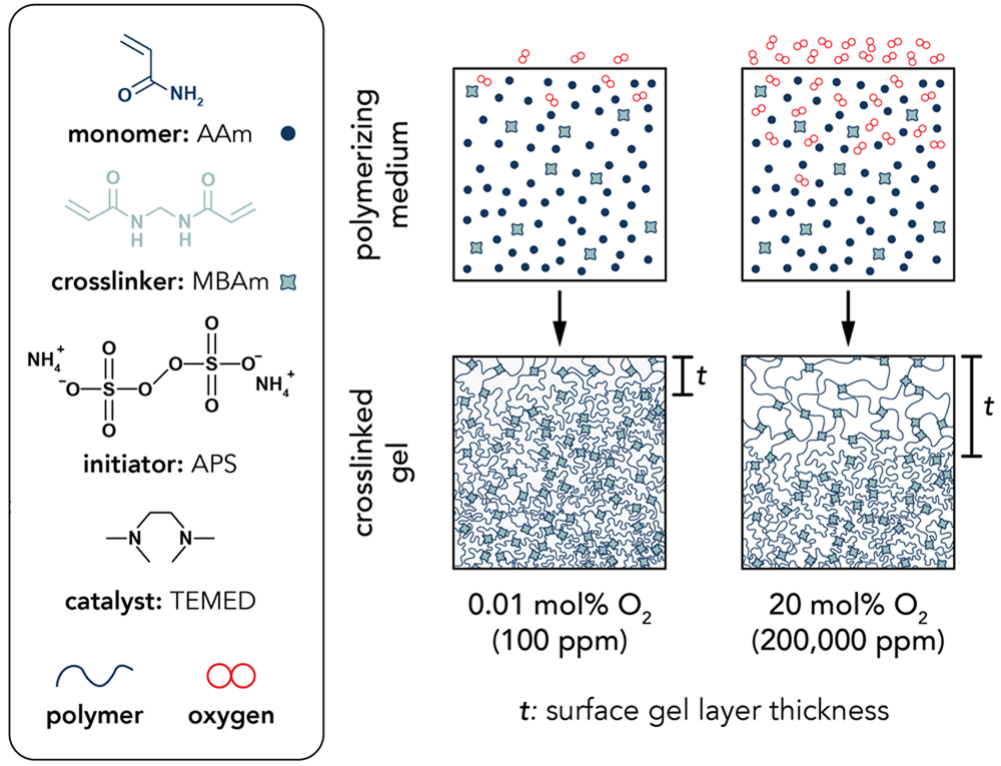
Chemical structures of the acrylamide (AAm) monomer (dark blue circle), *N*,*N*′-methylenebis(acrylamide) (MBAm) cross-linker (light blue cross), ammonium persulfate (APS) initiator, and *N*,*N*,*N*′,*N*′-tetramethylethylenediamine (TEMED) catalyst with schematics of the hydrogel structures after polymerization in 0.01 mol % O_2_ and 20 mol % O_2_. The surface gel layer thickness, *t*, is denoted.

For hydrogels synthesized via free-radical polymerization, the oxygen inhibition effect can result in a loosely cross-linked surface layer with a cross-linking and polymer concentration gradient.^59−61^ The thickness of this layer, denoted herein as the surface gel layer, is highly dependent on polymerization kinetics, cross-linker concentration, and oxygen diffusion and concentration (Figure 2).^59^ Pitenis et al. demonstrated the superlubricity (μ ≤ 0.005) of 7.5 wt % polyacrylamide hydrogels polymerized in ambient air (20 mol % O_2_) in self-mated gel-on-gel contact, although the surface layer was not characterized.^62^ Spencer and Simič controlled environmental oxygen (0–22% O_2_) during the polymerization of 9.6 wt % polyacrylamide hydrogels and demonstrated that with increasing oxygen, both the friction coefficient (μ ≈ 0.1 to μ ≈ 0.002) and elastic modulus (*E ≈* 21 kPa to *E* < 1 kPa) decreased.^63^ They postulated that these decreases could be explained by an increase in the thickness of the surface gel layer with increasing O_2_ content, although the thickness was not directly measured.

While these studies and many others have demonstrated that high water content surface layers are critical for achieving a lubricious sliding interface,^61,64−66^ it remains unclear what specific aspects of the surface gel layer structure (e.g., polymer concentration and thickness) tune the lubricity of hydrogels. The relationship between surface structure and properties is complex for heterogeneous hydrogels, and current experimental methods for estimating the surface gel layer thickness involve indirect techniques such as microindentation,^67,68^ nanoindentation,^42,61,63,66,69^ development of new contact mechanics models,^70,71^ neutron reflectivity,^72^ and confocal fluorescence microscopy.^37,59^ Further, experiments cannot reliably distinguish the interplay between a surface gel layer’s thickness, mesh size, polymer concentration, and concentration gradient (or lack thereof), particularly as they impact mechanical properties, such as lubricity and elastic modulus. While reaction–diffusion models for polymerization kinetics can predict these intricacies of the surface gel layer structure, no such models have been developed to estimate the surface gel layer thickness for polyacrylamide chemistry, including the effects of oxygen inhibition, despite the many kinetics investigations of photopolymerized thin film systems. In this work, a combined experimental and modeling approach provides a complete picture of the surface gel layer structure, mechanics, and tribology in 17.5 wt % polyacrylamide hydrogels synthesized in a controlled environment whose surface structure is tuned using oxygen concentration.

## 2 Materials and Methods

### 2.1 Hydrogel Synthesis

Acrylamide (AAm), *N*,*N*′-methylenebis(acrylamide) (MBAm), *N*,*N*,*N*′,*N*′-tetramethylethylenediamine (TEMED), and ammonium persulfate (APS) were purchased from Sigma-Aldrich. All of the reagents were used as received. Stock solutions of AAm (30 wt %), MBAm (2 wt %), TEMED (10 vol %, corresponding to 7.9 wt %), and APS (10 wt %) were prepared in ultrapure deionized (DI) water (18.2 MΩ·cm). Aliquots of each constituent were used to form a precursor solution of 17.5 wt % AAm and 0.7 wt % MBAm, following the methods by Urueña et al.^21^ The final TEMED and APS concentrations were 0.12 and 0.15 wt %, respectively. With a 54:1 molar ratio of monomer to cross-linker, most chain segments will incorporate at least one cross-linking group before reaching the entanglement molecular weight, which is 200 repeat units for 100 wt % polyacrylamide. Thus, polymerizing chains of sufficient length to entangle in our 17.5 wt % hydrogels are expected to have formed a cross-link instead; we anticipate potential entanglement effects to play a negligible role in our measurements.

The precursor solution (5 mL volume) was polymerized in a 35 mm diameter polystyrene mold, with the top surface exposed to the atmosphere as a “free surface”, creating an air–liquid interface. The prepolymerized solution height was 5.2 mm. Polymerization was completed in a glovebox, and an oxygen sensor (Alpha Omega Instruments, trace oxygen analyzer, Series 3520 G) with a range of 0–10,000 ppm of O_2_ was used to monitor the oxygen content within the glovebox. Humidity was controlled with a commercial portable humidifier, and the relative humidity and temperature were monitored with a thermohygrometer (Omega Digital Thermo-Hygrometer, Model #RH411). Gels were polymerized in two oxygen concentrations targeting 100 ppm of O_2_ (glovebox) and 200,000 ppm of O_2_ (atmospheric conditions), corresponding to 0.01 mol % O_2_ and 20 mol % O_2_, respectively, to alter the surface gel layer thickness (Figure 2). The reactions were quenched 15 min after the start of polymerization when the gels were submerged into a water bath at least 8 times the gel volume. Hydrogel sections (24 mm diameter) were equilibrated in DI water at room temperature in a sterile environment for at least 5 days before testing to ensure that equilibrium swelling was reached. We assume that oligomers that did not incorporate a cross-link prior to termination (i.e., free polymer) have been washed away by these steps and do not affect tribological results. For additional details on synthesis conditions, see SI Section 2.

### 2.2 Microindentation

**Figure 3:**
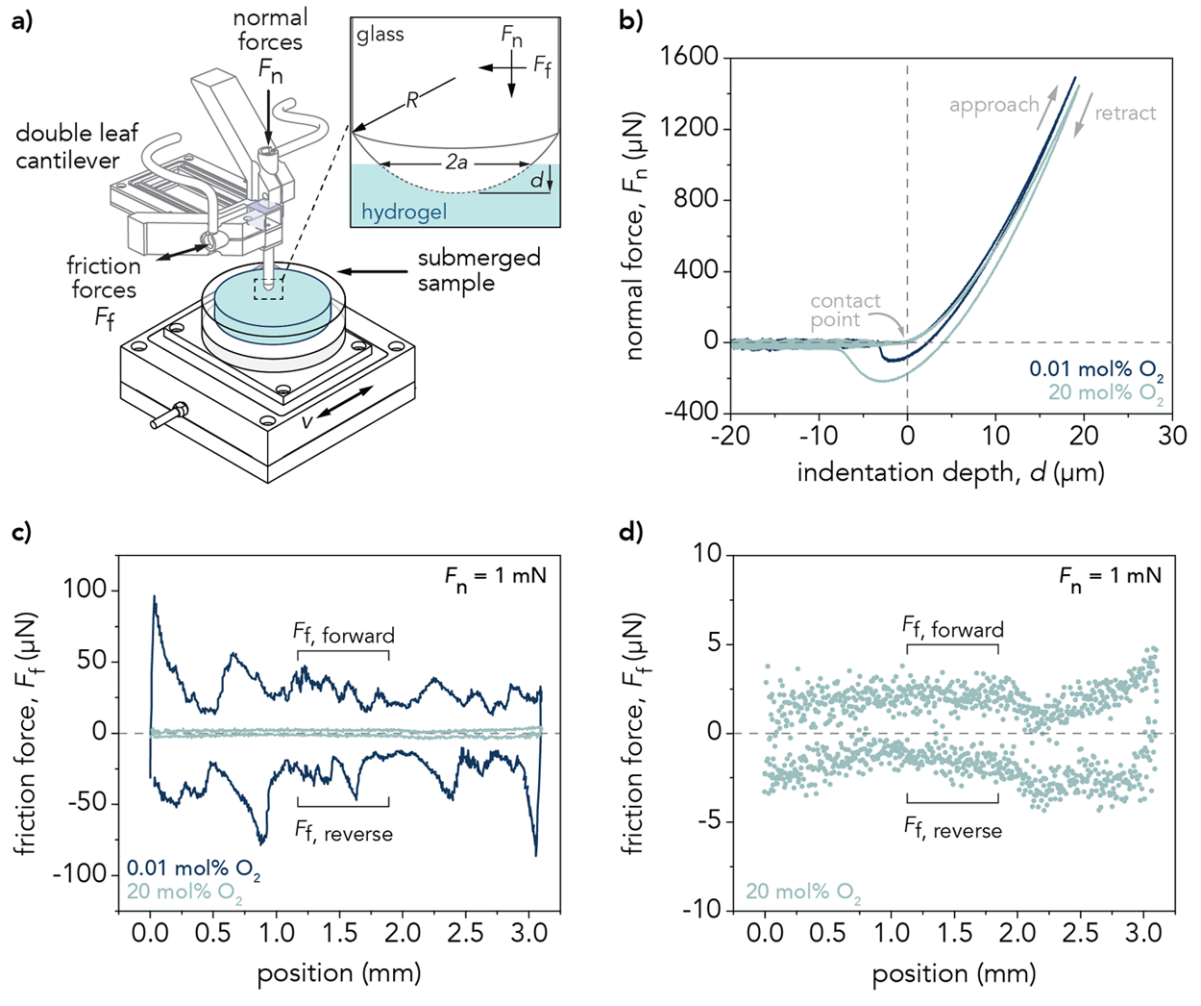
(a) Schematic of the linear reciprocating tribometer with inset demonstrating the contact diameter, 2*a*, between the glass probe with radius of curvature, *R*, and the hydrogel sample with an indentation depth, *d*. The applied normal forces, *F*_n_, and resulting friction forces, *F*_f_, are measured by capacitance probes, which convert the deflections of the double-leaf cantilever into forces. (b) Representative microindentation curves for the 17.5 wt % polyacrylamide hydrogels polymerized at 0.01 mol % O_2_ (dark blue) and 20 mol % O_2_ (light blue). The reduced elastic modulus, E*, is estimated by fitting the approach curves from the point of contact to *F*_n_ = 1 mN using Hertzian contact mechanics. (c) Representative friction force loops of the 17.5 wt % polyacrylamide hydrogels polymerized at 0.01 mol % O_2_ (dark blue) and 20 mol % O_2_ (light blue) at an applied normal force of *F*_n_ = 1 mN and sliding velocity of *v* = 0.5 mm/s. Friction coefficients for each cycle are calculated by analyzing the middle 25% of the sliding path. (d) Enlarged friction force loop of the hydrogel polymerized at 20 mol % O_2_ to highlight the extremely low friction forces.

Microindentations were conducted with a custom-built linear reciprocating tribometer to determine the reduced elastic modulus, E*, of the hydrogels (Figure 3a). A hemispherical borosilicate glass probe (radius of curvature, *R* = 3.1 mm) was mounted to a double-leaf cantilever flexure with normal and tangential spring constants of *K*_n_ = 221 μN/μm and *K*_f_ = 100 μN/μm, respectively. Capacitance probes measured the deflection of the cantilever (Lion Precision Elite Series, 5 μm/V sensitivity, 20 V range) and converted the displacement into forces. Indentation depth, *d*, was calculated by accounting for the compliance of the cantilever as *d* = *z* – *x*, where *z* is the vertical piezo displacement and *x* is the cantilever displacement. A maximum applied normal force of *F*_n_ = 1.5 mN was applied at an indentation velocity of 10 μm/s to three separate positions along the gel. Six indentation measurements were performed at each location, and the approach curves were fit up to *F*_n_ = 1 mN using Hertzian contact mechanics theory (eq 1) by minimizing the sum of squared errors to solve for E*:
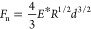
1where *R* is the probe radius of curvature, *d* is the indentation depth, and E* = *E*/(1 – *ν*^2^), where *E* is the compressive elastic modulus and ν is the Poisson’s ratio of the hydrogel. Representative indentation curves are displayed in Figure 3b. The reported elastic moduli are the averages and standard deviations of 54 total indentations spanning three separate gels. Representative Hertzian fits of the approach curve can be found in SI Section 3.

### 2.3 Nanoindentation

To characterize the stiffness of the surface gel layer, nanoindentations were performed by using an atomic force microscope (AFM, Asylum MFP-3D Bio). A spherical borosilicate colloidal probe (Novascan) with a radius of *R* = 2.5 μm and normal spring constant of *k* = 0.25 nN/nm was calibrated via indentations against a glass slide. Force curves were generated at varying normal forces (*F*_n_ = 2–400 nN) with an indentation velocity of 2 μm/s. Measurements were completed at 25 °C with the gels submerged in DI water. Hertzian contact mechanics (eq 1) was used to estimate the reduced elastic modulus, E*, of the surface gel layer by fitting the approach curves up to an indentation depth of 0.55 μm, determined by the small-angle approximation theory and probe radius of curvature (SI Section 3.3). This small indentation depth also ensured that the analysis stayed within the strains allowable for Hertzian contact mechanics.^73^ The reported elastic moduli are the averages and standard deviations of 15 total indentations spanning three separate gels and 10 indentations spanning two separate gels for the 0.01 mol % O_2_ and 20 mol % O_2_ gels, respectively.

### 2.4 Friction Measurements

Tribological experiments were conducted with a linear reciprocating tribometer. Hydrogel friction coefficients were obtained with a hemispherical borosilicate glass probe (radius of curvature, *R* = 3.1 mm) mounted to a double-leaf cantilever with normal and tangential spring constants of *K*_n_ = 221 μN/μm and *K*_f_ = 100 μN/μm, respectively. While the samples were fully submerged in DI water, *F*_n_ = 1 mN (contact pressure, *P* ≈ 9–11 kPa) was applied at a sliding velocity of 0.5 mm/s for a sliding path length of 3 mm. The normal and friction forces were averaged over the middle 25% of the sliding path to calculate the cycle friction coefficient, μ_cycle_, using eq 2:
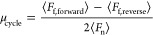
2where *F*_f,forward_ and *F*_f,reverse_ are the friction forces in the forward and reverse direction, respectively, and *F*_n_ is the applied normal force. The sample friction coefficient, μ_sample_, was obtained by averaging μ_cycle_ over 30 cycles. The reported friction coefficients are the averages and standard deviations of μ_sample_ values across three separate gels. The theoretical noise floor for the friction coefficient measurements is μ_min_ = 0.0005 (SI Section 4). Representative friction force loops are displayed in Figures 3c and 3d. For additional details regarding contact area and pressure calculations, see SI Section 5.

### 2.5 Model Development

**Scheme 1:**
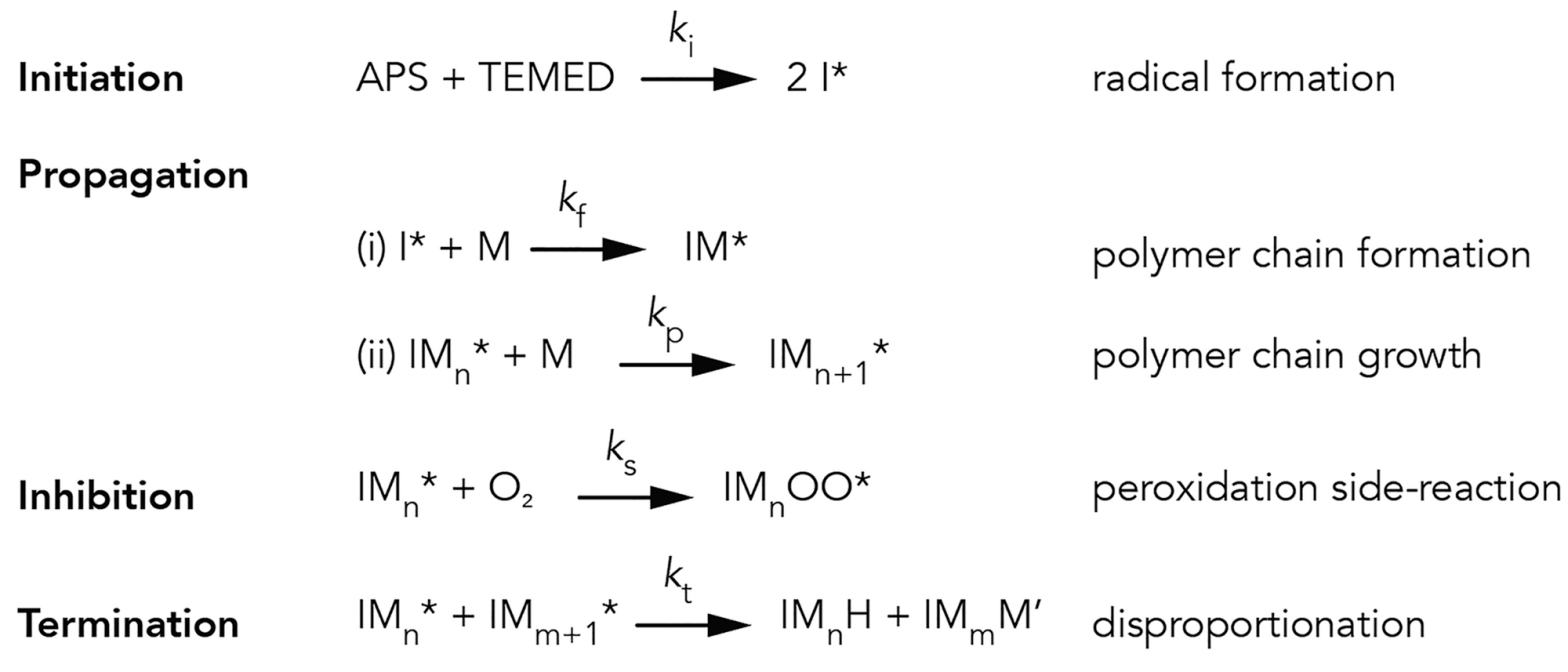
Reaction Mechanism of a Redox-Initiated Free-Radical Polymerizing System In the present case, APS and TEMED are the initiator and catalyst, respectively. The rate constant, *k*, for each step is denoted with a subscript. *M* represents a reactive unit, i.e., acrylamide or *N*,*N′*-methylenebis(acrylamide) monomers or cross-linkers, respectively, M* is the oligomer-bound radical, O* is the oxygen radical, and I* is any possible initiation species. The dominant termination mechanism in polyacrylamide is not biomolecular termination but rather disproportionation,^74,75^ in which one chain-terminal radical reacts with the hydrogen (*H*) neighboring a second chain-terminal radical to form a hydrogen-terminal chain and a new double bond (*M′*).

Because the entire set of reaction mechanisms associated with polyacrylamide polymerization is known (Scheme 1), a reaction–diffusion model that accounts for the precise geometry and gel component concentrations used herein (Section 2.1) can be generated. Using reported values for reaction constants, this model is quantitative, without any adjustable parameters. Several analytical models have been put forth to understand the effect of oxygen inhibition in photopolymerizations of various acrylate monomers in thin films^45,55^ or microfluidic geometries^47^ and in other chemistries.^57^ Despite the ubiquity of acrylamide polymerization in applications as commonplace as gel electrophoresis, no similar models have been developed for acrylamide chemistry. Additionally, prior modeling efforts have not investigated the free surface of the gel with oxygen inhibition in any chemistry or geometry.

Our model builds on extensive prior work on free-radical polymerization with peroxidation side-reaction.^45,47,55,57^ The polymerization is initiated by consumption of APS and TEMED, which produce radicals at a rate of initiation (*r*_i_), given by
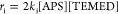
3In the second step, the primary radicals react with an unconverted double bond (*M*), either on acrylamide or *N*,*N*′-methylenebis(acrylamide) monomers, and continue to propagate in chain growth at the rate *r*_p_:
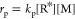
4In this expression, R* represents all reactive radicals, which are I*, IM*, and IM_n_*; in other words, we assume all radicals are equally reactive (*k*_f_ ≈ *k*_p_) as well as equivalent reactivity between AAm and MBAm monomers when reacting with growing polymer chains. Note that the IM_n_OO* radicals that form via the peroxidation side reaction are stable and do not participate further in the reaction after formation. Reactive radicals can also be consumed by various classical termination mechanisms. Here, we consider only termination by disproportionation, whose prevalence in polyacrylamide polymerization greatly surpasses all other termination modes, including conventional biomolecular termination.^74,75^ In sum, the total rate of radical termination (*r*_t_) from inhibition and termination is given by
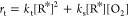
5

In the real system, MBAm is more reactive than AAm, causing early formation of MBAm-rich sequences that act as more compact subdomains in the gel as the composition of residual monomer drifts to further favor AAm as the reaction proceeds.^76^ This and other causes of molecular-level inhomogeneity, such as statistical chemical heterogeneity, are excluded from the present model for simplicity. While the assumption of acrylamide and bis(acrylamide) reactive equivalence is limited, the local structure of the monomers is similar: the bis(acrylamide) simply has two separate reactive groups that react independently with either oxygen or other moieties; each end reacts in a first-order manner with the same mechanism as the acrylamide monomer.^77^Figure S3 and the associated discussion in the Supporting Information provide in-depth details of the diversity of structures formed during polymerization. While kinetic constants for oxygen reactivity with bis(acrylamide) and its substituents have not been measured, the polymerization of both AAm and MBAm will be inhibited more at higher local oxygen concentrations in the real system. This is the main effect our model aims to probe, and its microscale impacts are captured without differentiating AAM and MBAm reactivity individually.

To develop the model equations, we seek an expression for the monomer conversion, *X*, at various depths *z* in the polymerizing medium and as a function of reaction time *t*, . Monomer conversion reveals the progress of the gelation reaction at every depth from the surface (*z* = 0) to the bottom of the casting mold (*z* = *H*). The rate of monomer consumption, , is equivalent to *r*_p_. Therefore, to extract *X*(*t*,*z*), we require an expression for [R*] in terms of known variables—reaction constants and initial concentrations of [APS]_init_, [TEMED]_init_, and [O_2_]_init_ (see Tables S5 and S6)—as well as variables accessible by differential equations: the time-dependent concentrations of APS, TEMED, and O_2_. We assume that the bottom and sides of the gel casting mold are impermeable to oxygen so that net diffusion of oxygen is symmetric in *x–y*. Thus, reactivity is a function of depth only, and the model is one-dimensional. In addition to the rate laws above, achieving the expression for [R*] requires a species balance describing the reaction–diffusion of oxygen in the *z*-direction into the polymerizing medium:
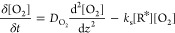
6The dissolved oxygen content at the upper surface of the reacting medium is assumed to remain in equilibrium with the surrounding atmosphere throughout the polymerization for each O_2_ concentration (0.01 and 20 mol % O_2_). We estimate this constant dissolved oxygen content at the upper surface using Henry’s law constant *H*^cc^ = 0.032 at standard temperature and pressure for oxygen dissolution into water.

The mathematical steps and nondimensionalization required to derive *X*(τ,η), the final expression for monomer conversion as a function of dimensionless depth, , and time, , are provided in SI Section 8. The solution assumes a net zero production rate of radical species, i.e., that R* are consumed as soon as they are generated and that the temperature remains constant so that the rate constants and oxygen diffusivity, *D*_O_2__, are constant throughout the reacting medium. *X*(τ,η) is given by
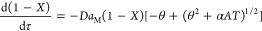
7where  is the dimensionless oxygen concentration, *A* and *T* are the dimensionless APS and TEMED concentrations,  is a dimensionless group representing the rate of initiation of the polymerization relative to the rate of oxygen side-reactions, and  is the monomer Damköhler number that relates the reaction and diffusive transport rates of the monomer. Evaluating this expression requires solutions for θ, *A*, and *T* as the functions of τ and η. APS and TEMED concentrations are solved using the coupled differential equations:
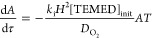
8
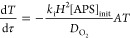
9θ(τ,η) is obtained by the nondimensionalized reaction–diffusion equation for O_2_:
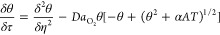
10where  is the oxygen Damköhler number.

## 3 Results and Discussion

In this work, 17.5 wt % polyacrylamide hydrogels were polymerized at two oxygen concentrations (0.01 and 20 mol % O_2_) with one surface exposed as a “free surface” to the atmosphere to create hydrogels with surface gel layers of varying depth. Polymerization of this system occurred at the air–liquid interface, and it was expected that the hydrogels exposed to higher oxygen concentration during polymerization would have thicker gradient surface gel layers and therefore lower friction coefficients, *μ*, and reduced elastic moduli, E*. Tribological sliding experiments were performed to measure the friction coefficients, and an analytical model based on polymerization kinetics was developed to estimate the surface gel layer thickness and its gradient. Nanoindentation was conducted to determine the surface mechanics accompanied by microindentation to probe deeper within the surface gel layer.

### 3.1 Friction Coefficients

The average friction coefficients for the 17.5 wt % polyacrylamide hydrogels were calculated over 30 sliding cycles at an applied normal load of *F*_n_ = 1 mN (contact pressure, *P* ≈ 9–11 kPa) and a sliding velocity of *v* = 0.5 mm/s and averaged across three separate gels. Smooth, hemispherical glass probes were used as the countersurface. For gels prepared at 0.01 mol % O_2_, the friction coefficient was μ = 0.021 ± 0.006. In contrast, the friction coefficient was an order of magnitude lower (μ = 0.002 ± 0.001) for samples prepared at 20 mol % O_2_, which is within the superlubricity regime (μ ≤ 0.005) (Figure 3c,d). Low friction was maintained for these samples, and the friction coefficients did not significantly change over the duration of the experiment (see SI Section 10 and Figure S5), suggesting that the surface gel layer had sufficient time to recover before the next reciprocating cycle. If compression and local draining of the surface gel layer had occurred, we would have expected the friction coefficient of 20 mol % O_2_ hydrogels to gradually increase over time to values similar to those exhibited by 0.01 mol % O_2_ hydrogels.

The drastic reduction in friction coefficient by simply increasing the oxygen concentration during polymerization while maintaining the same initial monomer and cross-linker concentrations was also observed by Simič and Spencer with 9.6 wt % polyacrylamide gels cast against polyethylene membranes in varying oxygen concentrations (0–22% O_2_).^63^ In self-mated gel-on-gel contact, the friction coefficient decreased from μ ≈ 0.1 at 0% O_2_ to μ ≈ 0.006 at 22% O_2_ at a sliding speed of *v* = 0.5 mm/s and contact pressure *P* ≈ 6–10 kPa.^63^ For the gels at 22% O_2_, the authors estimated the thickness of the surface gel layer to be greater than 15 μm based on non-Hertzian responses during nanoindentations. This decrease in friction with increasing oxygen concentration suggests that either the surface gel layer increased in thickness or potentially had a more loosely cross-linked structure at the surface, or some combination thereof.

Comparisons against other polyacrylamide hydrogels ranging in monomer (2.25–17.5 wt %) and cross-linker (0.03–0.7 wt %) concentrations (see Table S7 and Figure S4) also highlight the extremely low friction coefficients exhibited by our 17.5 wt % polyacrylamide hydrogels, despite high polymer concentrations and the use of an impermeable glass probe as the countersurface as opposed to another gel. Dunn et al. demonstrated that self-mated gel-on-gel sliding led to lower friction coefficients than glass-on-gel for 8 wt % polyacrylamide hydrogels with 0.5 wt % MBAm cross-linker at low sliding speeds (*v* = 0.1 mm/s), but this distinction lessened at higher sliding speeds.^78^ They demonstrated that glass-on-gel friction coefficients were dominated by continuous polymer–glass interactions, while the probability of polymer chain interactions in the gel-on-gel configuration was greatly reduced, leading to greater solvation and lower friction coefficients. Therefore, it is expected that sliding experiments conducted with glass probes as the countersurface would have larger friction coefficients. Yet, the friction coefficients measured herein for the hydrogels at 20 mol % O_2_ are remarkably low, even lower than the friction coefficients for self-mated 3.75 wt % polyacrylamide hydrogels (μ = 0.005),^21^ which could be due to the absence of a molding surface because our hydrogel surfaces were exposed to the atmosphere and polymerized at this air–liquid interface.

Tribological sliding experiments were also conducted at varying normal loads (Figure S6) and sliding speeds (Figure S7) to further investigate the tribological behavior of the hydrogels. For self-mated gel-on-gel sliding configurations, friction coefficients typically decrease with increasing load due to nonlinear contact radius scaling.^79,80^ Friction coefficients also tend to increase with sliding velocity past a critical transition velocity, v*, due to the inability of the hydrogel to relax shear strain.^21,80^ The 17.5 wt % polyacrylamide gels herein deviated from previously observed tribological behavior—for the gels at 0.01 mol % O_2_, μ slightly increased with increasing load (Figure S6a); for both oxygen conditions, μ decreased with increasing sliding speed (Figure S7). Shoaib et al. similarly observed decreasing μ with increasing *v* when below v*, indicating that faster sliding velocities may be required to surpass v* for these gels.^81^ While further experimentation is warranted to understand these interesting tribological behaviors, they may be indicative of poroelastic effects due to the complex gradient surface gel layer. We leave the investigation of this complex behavior to future studies.

### 3.2 Microindentation Measurements and Analysis

The reduced elastic modulus of the top 15 μm of the polyacrylamide hydrogel samples was obtained with microindentations along three different positions and averaged across three independent hydrogel samples at a constant indentation velocity of 10 μm/s. At 0.01 mol % O_2_, E* = 242 ± 11 kPa, with a slight decrease to E* = 203 ± 38 kPa at 20 mol % O_2_. Representative indentation curves and Hertzian contact mechanics fits can be found in Figures 3b and S1. At first glance, the slightly higher modulus reported for the 0.01 mol % of O_2_ could be explained by less peroxyl formation in the gel surface with dissolved oxygen at the start of the reaction leading to smaller predicted mesh sizes, as shown by the model in Section 3.4. However, there is no statistical significance between the experimentally determined E*. Our results suggest that the modulus of hydrogel surfaces at large indentation depths is insensitive to differences in oxygen content in real systems and is largely determined by the starting concentrations of reactant species; in our experiments, the starting dissolved oxygen concentration even in the 20 mol % O_2_ case was an order of magnitude smaller than the APS (initiator) and TEMED (catalyst) concentration.

To examine the effects of indentation velocity on the measured elastic modulus, the hydrogels were indented at a lower velocity, *v* = 1 μm/s, which was comparable to the velocity used during nanoindentations (Section 3.3 and Figure S8). The indentation velocity did not significantly impact the elastic modulus measurement of the 0.01 mol % O_2_ hydrogels, but the measured elastic modulus decreased by about 18% for the 20 mol % O_2_ hydrogels. Our results suggest poroelastic behavior due to the presence of a highly hydrated surface gel layer.

### 3.3 Surface Mechanics

**Figure 4:**
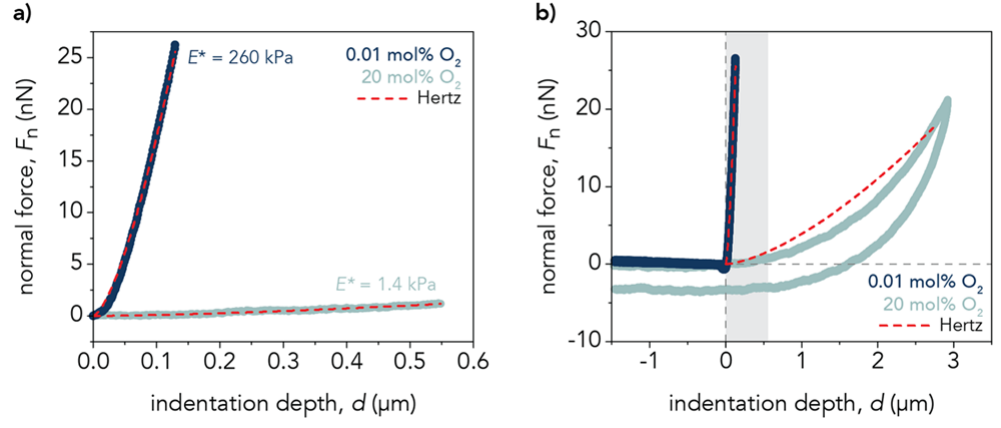
(a) Representative nanoindentation approach curves for the 17.5 wt % polyacrylamide hydrogels at 0.01 mol % of O_2_ (dark blue) and 20 mol % of O_2_ (light blue). Hertzian contact mechanics (red dotted line) was used to fit the first 0.55 μm (gray region) of the approach curve to determine the reduced elastic modulus, E*. For the plotted curves, E* = 260 kPa and E* = 1.4 kPa at 0.01 mol % O_2_ and 20 mol % O_2_, respectively. (b) Full nanoindentation curves for the hydrogels demonstrating the deviation of the Hertz contact model past 0.55 μm for the hydrogel at 20 mol % O_2_.

Atomic force microscopy (AFM) was used to probe the mechanics of the superficial region of the surface gel layer across two to three independent hydrogel samples at a constant indentation velocity of 2 μm/s. Only the first 0.55 μm of the approach curve was analyzed with Hertzian contact mechanics to stay within the small-angle approximation and allowable strain (SI Section 3.3). Figure 4 displays representative nanoindentation approach curves for the polyacrylamide hydrogels cast at 0.01 mol % O_2_ and 20 mol % O_2_. Across three independent gel samples, the average reduced elastic modulus at 0.01 mol % O_2_ was E* = 257 ± 48 kPa. At 20 mol % O_2_, E* = 1.1 ± 0.2 kPa across two independent hydrogel samples. For additional representative nanoindentation curves, see Figure S9.

The low reduced elastic modulus value for the hydrogels cast in 20 mol % O_2_ indicates that the gel structure at the top 0.55 μm of the hydrogel surface is extremely soft. In both cases, the surface is directly exposed to the atmosphere as a “free” surface in local equilibrium with atmospheric oxygen and not cast against any solid material. This may explain why our 17.5 wt % polyacrylamide hydrogels with 0.7 wt % cross-linker cast in 20 mol % O_2_ have similar reduced elastic moduli at the surface to gels in the literature cast against polyethylene membranes in 20 mol % O_2_ (*E* < 1 kPa as measured by AFM), although these previously reported gels have *lower* polyacrylamide and cross-linker concentration at 9.6 and 0.4 wt %, respectively.^63^ The 17.5 wt % polyacrylamide hydrogel surfaces cast herein at an air–liquid interface may be more loosely cross-linked than the literature reports for polyacrylamide hydrogels cast against molding surfaces despite higher polymer concentrations in the bulk (see Table S7).^21,41,42,61,63,66−70,82^ These results highlight that the surface effects of oxygen inhibition are best observed and exploited at a free surface where oxygen can easily diffuse throughout the duration of the reaction, as demonstrated in more detail in Section 3.4. Moreover, increasing the oxygen concentration results in a significantly different surface structure despite having a minimal effect on the mechanical properties of the gel at larger indentation depths.

Owing to its simplicity, the Hertz contact mechanics model has been broadly deployed to characterize heterogeneous materials, such as articular cartilage,^83^ cells,^84^ and hydrogels.^63^ At low indentation depths within the small strain limit (≤0.55 μm), the Hertzian contact model fits the data well (Figure 4a). However, the fit deviates from the approach curve at 20 mol % O_2_ at higher indentation depths, indicating that this model may not be the most appropriate one to use (Figures 4b and S10). Because the Hertz model assumes a homogeneous network, the observed deviations support heterogeneity of the network at the surface. Johnson and Dunn similarly observed non-Hertzian behavior for polyacrylamide hydrogels of varying concentrations cast against polystyrene in ambient conditions and used the Fredrickson high-penetration brush model and Winkler contact mechanics model to fit the initial portions of the indentation curves to estimate the thickness of the surface gel layer.^67,69^ For our 20 mol % O_2_ hydrogels, the Fredrickson high-penetration brush model fit the entirety of the nanoindentation approach curve (Figure S10), suggesting a thick surface gel layer.

### 3.4 Modeling Gelation at the Surface

We postulate that the measured frictional decrease and lower surface elastic modulus for the hydrogels polymerized at 20 mol % O_2_ compared to those at 0.01 mol % O_2_ can be attributed to a thicker surface gel layer with larger mesh size. However, the relative impacts of oxygen content on surface gel layer thickness, polymer concentration, and cross-link density are difficult to distinguish in any further detail using experiments. Here, we developed a reaction–diffusion model for conversion of acrylamide and cross-linker monomers under various conditions as the polymerization progresses, both in the bulk and at the surface of the gel. We used this model to gain insight into the formation of the surface gel layer under the tested conditions as well as its structure after 15 min of polymerization at 0.01 and 20 mol % O_2_. This model predicts conversion of monomers to polymers as a function of time and depth from the gel surface, which corresponds to the concentration of polymer chains in the gel prior to swelling.

Because AAm and MBAm are treated equivalently in the model (see Section 2.5), the predicted cross-link density is directly proportional to the predicted monomer conversion. However, in the experimental system, bis(acrylamide) radicals are much more reactive with both acrylamide and bis(acrylamide) monomers than are acrylamide radicals.^18,76^ The enhanced incorporation of bis(acrylamide) monomers at early reaction times in the real system leads to the well-known microscale heterogeneity of the polyacrylamide network, with formation of microdomains of large segmental density.^85^ Our model cannot capture this heterogeneity due not only to the equivalence assumption discussed above but also because the composition is treated as a continuum across every calculated depth within the gel. Thus, while our model predictions suggest trends in cross-link density, we restrict our interpretation to average polymer concentration per depth from the surface in the preswollen gel.

Our model intends to provide a micrometer-scale description for gel structure from the surface to the gel bulk via the polymer concentration at each depth rather than segmental-level structural detail. Therefore, we describe the observed trends in terms of mesh size to be purposefully agnostic to the molecular structure at the surface of the gel. For example, both a “brushy” structure such as that postulated by Simič et al.^42^ and one with very long connective chains between cross-links could have a large mesh size. Regardless, our results demonstrate that considering 10s to 100s μm of gel layer thickness at the surface is important because the oxygen conditions during polymerization affect polymer concentrations throughout this entire depth, which in turn governs the mechanical and tribological gel properties; any segmental-scale mechanistic explanations for surface polymer conformation in the lower friction oxygen-inhibited gels must be compatible with the reduced concentration of polymer at the gel surface predicted herein.

#### 3.4.1 Gelation at Low Oxygen Concentration

**Figure 5:**
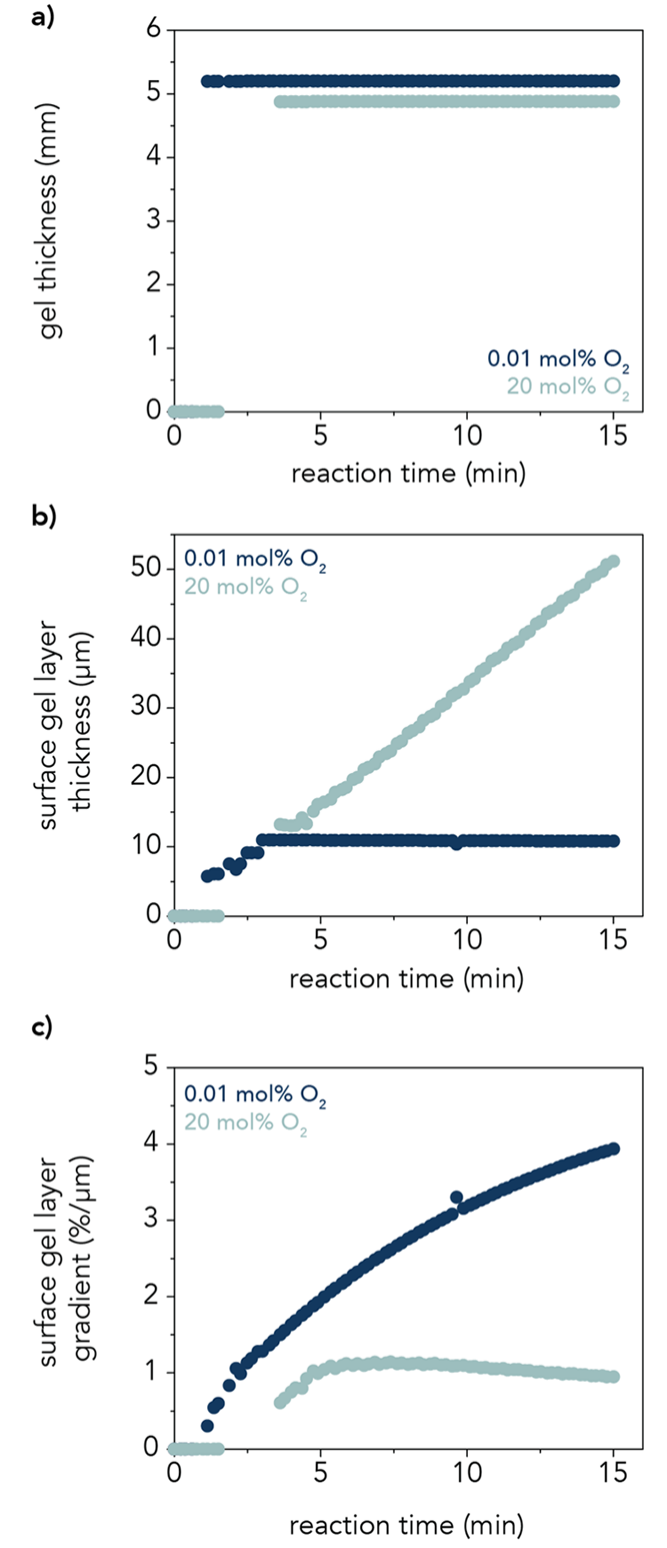
Predictions of the reaction–diffusion model under low and high oxygen conditions. (a) Bulk gel formation occurs within minutes of adding APS and TEMED to initiate the polymerization, reaching a thickness of 5.2 mm for the 0.01 mol % O_2_ hydrogels (dark blue) and 4.9 mm for the 20 mol % O_2_ hydrogels (light blue). (b) Surface layer thickness, extrapolated from fits of the monomer conversion in the surface gel layer as a function of polymerization time. At 0.01 mol % of the O_2_ (dark blue), the surface gel layer thickness slowly decreases with reaction time after its initial formation as the gel polymerizes. At 20 mol % O_2_ (light blue), the surface gel layer thickness increases throughout the polymerization. (c) Severity of gradient in monomer conversion from the surface of the gel to the bulk increases with polymerization time at 0.01 mol % O_2_ (dark blue) but decreases slightly with time at 20 mol % O_2_ (light blue). At 15 min polymerization, the surface gel layer conversion gradient is 4%/μm and 1%/μm at 0.01 mol % and 20 mol % O_2_, respectively.

**Figure 6:**
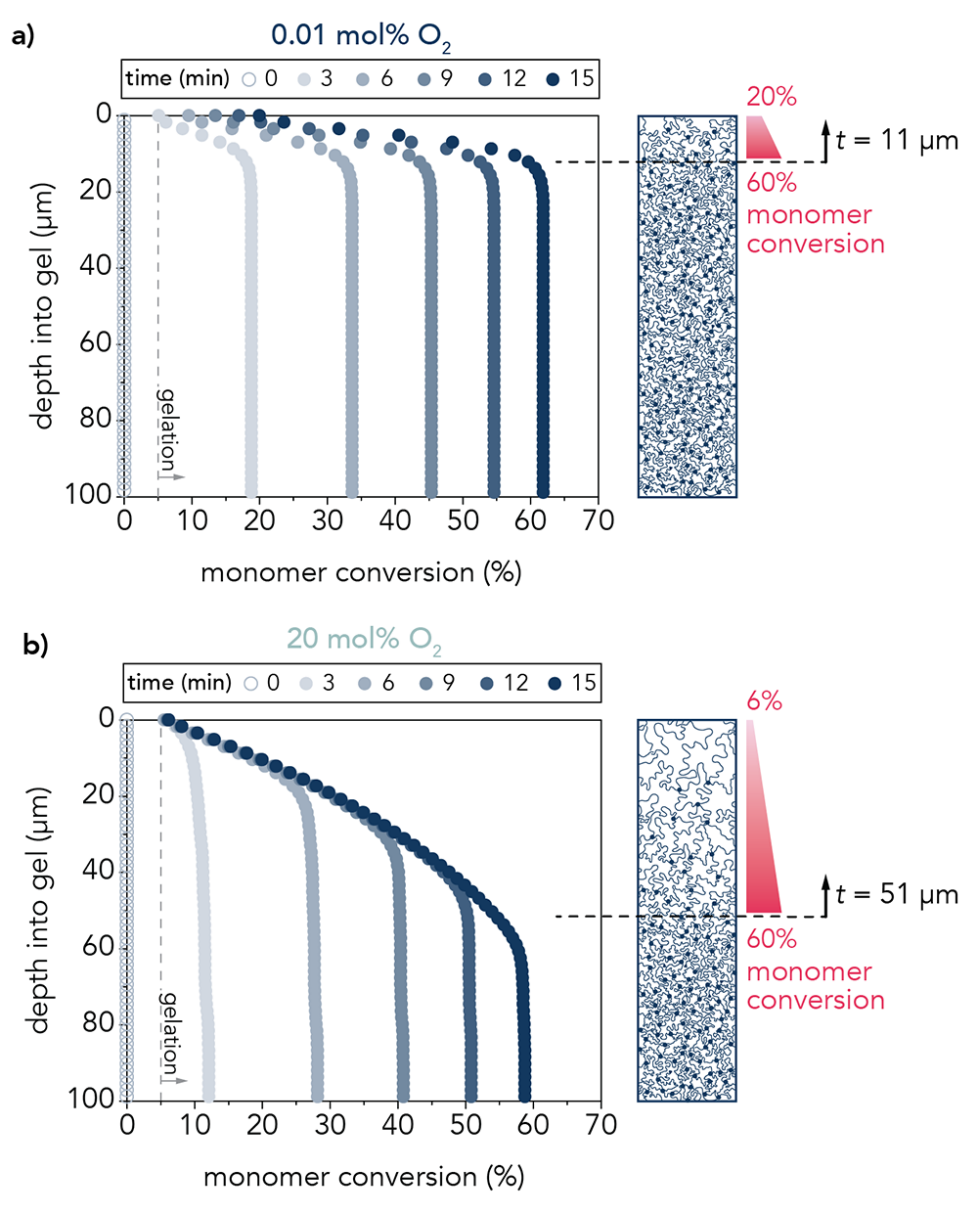
Monomer conversion at the surface of the gel at various polymerization times as a function of depth from the gel surface for the (a) 0.01 mol % and (b) 20 mol % O_2_ hydrogels. Gelation occurs around 5% monomer conversion. At the surface of the 20 mol % O_2_ hydrogels, conversion remains just above the gel point at 6%. Schematic representation of the top 100 μm of the hydrogel structures at 0.01 mol % O_2_ and 20 mol % O_2_ demonstrates that the surface gel layer is thicker with a smaller monomer conversion gradient at higher O_2_ compared to the surface gel layer at lower O_2_. This indicates a more gradual transition in polymer concentration from the surface gel layer to the bulk at 20 mol % O_2_, contrasting a more abrupt change in polymer concentration at 0.01 mol % O_2_.

During gelation at low oxygen concentration (0.01 mol % O_2_), predicted monomer conversion proceeds uniformly throughout the depth of the gel, apart from the surface where oxygen continues to diffuse into the polymerizing medium (Figure S11). Consequently, dissolved oxygen is locally enhanced near the surface and reacts to form peroxides at an elevated rate. As the reaction proceeds, a gel is predicted to form quickly (Figure 5a), along with a surface gel layer whose distinction from the bulk gel becomes clearer over time. At this O_2_ concentration, the thickness of the surface gel layer remains approximately constant once developed (Figure 5b). Meanwhile, the gradient in monomer conversion (i.e., polymer concentration) from the surface of the gel to the bulk—as given by the slope of the conversion in the surface gel layer—becomes steeper with time (Figure 5c) because progression of the polymerization at the surface cannot keep up with the rate of conversion in the bulk. The methodology for determining the point of gelation based on monomer conversion, the surface of the gel, and extracting surface layer thickness and gradient from the monomer conversion traces is provided in SI Section 13. The predicted apparent surface gel layer is very thick (>50 μm) early in the gelation process because the distinction between the surface layer and bulk gel is ill-defined at very early times. This indistinction is reflected in a larger error associated with the fit used to extract the surface layer thickness (Figure S13). However, two distinct regions are quickly established as the bulk gel formed, and the surface gel layer thickness quickly decreases to approximately 10 μm, after which it remains almost constant. As polymerization proceeds past 5 min, the surface gel layer thickness decreases slightly (Figure S14a), and its gradient becomes more severe. The conversion of monomer to polymer narrowly outcompetes the peroxidation side-reaction from dissolved oxygen at the gel’s surface, whereas in the gel bulk—where the oxygen concentration remains negligible after the first few minutes of the reaction— polymerization strongly dominates any peroxidation. After 15 min of polymerization, a surface gel layer 10.8 μm thick with a monomer conversion gradient of approximately 4%/μm is predicted, compared to a total gel thickness of 5.2 mm. Thus, before post-polymerization swelling, the polyacrylamide hydrogel samples polymerized at 0.01 mol % O_2_ are predicted to have an ≈11 μm thick surface gel layer, and the polymer concentration at the surface is about three times less than the bulk (Figure 6a) with a corresponding decrease in cross-link density.

#### 3.4.2 Gelation at High Oxygen Concentration

During gelation at higher ambient oxygen conditions (20 mol % O_2_), predicted monomer conversion remains negligible at the surface of the polymerization medium due to significant oxygen inhibition of the polymerization. This upper layer never forms a gel and is presumably washed away when gels are plunged into water to quench the polymerization, causing the hydrogel to be about 325 μm thinner than the gel at 0.01 mol % O_2_ (Figure S15). This unpolymerized liquid layer was similarly observed in studies of oxygen inhibition of acrylate thin films.^46,47,49,55^ Below this ungelled film, the polymerization proceeds to reach conversions above 5%, forming a gel as soon as the oxygen is consumed (Figure S12). Like the 0.01 mol % O_2_ case, oxygen consumption in the bulk takes about 2 min, after which a gel forms throughout the system (Figure 5a). As the polymerization proceeds, the distinction between the surface gel layer and bulk gel takes longer to establish than at 0.01 mol % O_2_, but unlike the low oxygen case, its thickness continues to increase significantly with polymerization time once the distinction between layers is established (Figure 5b). At every time point after initial formation of the surface gel layer (≈ 2.5 min), the thickness of this layer is larger for 20 mol % O_2_ than for 0.01 mol % O_2_. Meanwhile, the severity of the gradient in monomer conversion in the surface gel layer remains relatively constant once it is established (Figure 5c), and it even decreases slightly according to fits of the monomer conversion at the surface of the gel (Figure S14b). After 15 min, a 51.2 μm thick surface gel layer is predicted compared to a total gel thickness of 4.9 mm, with a 6.2% monomer conversion at the surface of the gel and a surface gel layer monomer conversion gradient of 1%/μm (Figure 5c). The surface polymer concentration is almost 10 times less than the bulk, whose monomer conversion is already slightly lower than that of the 0.01 mol % O_2_ case, consistent with the small experimentally observed decrease in E* of the hydrogel (Figure 3b).

#### 3.4.3 Comparison of the Surface Gel Layer Profiles

The surface gel layer thickness predictions herein for the 17.5 wt % polyacrylamide hydrogels cast in 20 mol % O_2_ are within the same order of magnitude as those predicted by indentations and contact mechanics modeling for polyacrylamide hydrogels ranging in monomer (7.5–15 wt %) and cross-linker (0.03–0.6 wt %) concentrations (Table S7).^21,41,42,61,63,66−70,82^ However, they are larger than previous estimates for the highest concentration hydrogel (15 wt % AAm, 0.6 wt % MBAm), which estimated a surface gel layer thickness of 13 μm for a hydrogel cast against a polystyrene surface in ambient air (20 mol % O_2_),^69^ as compared to our estimate of 51 μm. Gombert et al. used neutron reflectometry and infrared spectroscopy to compare the surface structures of 7.5 wt % polyacrylamide hydrogels cast against glass and PDMS surfaces in extremely low oxygen environments (< 0.01 mol % O_2_).^61^ They determined that a polymer concentration gradient formed for the gels cast against PDMS due to its hydrophobicity and predicted that this gradient would extend to more than 50 μm. This may indicate the importance of an air–liquid interface for the formation of thick surface gel layers for high polymer concentration hydrogels.

Previous studies did not resolve the polymer concentration gradient within the surface gel layer. However, the model herein predicts the conversion of monomers to polymers, which corresponds to the concentration of polymer chains in the gel prior to swelling. Over the course of polymerization, the severity of the monomer conversion gradient from the surface to the bulk increases with time for both oxygen concentrations (Figure 6). However, monomer conversion at the surface of the 0.01 mol % O_2_ gels increased, while conversion remained just above 5% at 20 mol % O_2_. During hydrogel polymerization at 20 mol % O_2_, the monomer conversion profile in the surface layer remains constant in time, whereas the bulk increases in conversion over time. Because oxygen is progressively consumed as it diffuses further into the film, a higher conversion is reached as less oxygen inhibition occurred deeper into the polymerizing medium. This effect is not observed at 0.01 mol % O_2_ because the oxygen concentration was low enough that it is entirely consumed at short depths into the gel, essentially forming a reaction–diffusion boundary layer. There is a steeper transition from the surface polymer concentration to the bulk over 11 μm (4%/μm) in comparison to the 20 mol % O_2_ case in which the surface gel layer polymer concentration transitioned more gradually at 1%/μm over 51 μm (Figure 6), leading to a surface gel layer that is approximately 5 times thicker and 4 times less dense than the one at 0.01 mol % O_2_ prior to swelling. While these conclusions assume that overall monomer conversion is related to polymer concentration, the large decrease in friction coefficient achieved in gels prepared at 20 mol % O_2_ support our predictions that the 0.01 mol % O_2_ and 20 mol % O_2_ gels have entirely different surface characteristics. In particular, our predictions show that not only a thicker surface gel layer but also a combination of a thicker layer and less concentrated polymer at the surface structure give rise to superlubricity in polyacrylamide gels polymerized at higher oxygen concentrations.

Overall, the model predicts qualitatively different structures and time-dependent formation behavior of the surface gel layer when the liquid–air interface is maintained at low versus high O_2_. In Figure 6, we observe two shapes of the surface gel layer as the polymerization proceeds in time. For 0.01 mol % of O_2_ at the interface, the model predicts that oxygen depletion in the bulk leads to the formation of a reaction–diffusion boundary layer at the air–liquid surface, resulting in a surface gel layer whose thickness remains relatively constant in time after its initial formation but with a gradient that sharpens over time due to the increasing conversion in the bulk gel. By contrast, for 20 mol % O_2_ at the interface, the model predicts a gradient that remains relatively static but penetrates deeper into the gel as oxygen diffuses further, resulting in a thicker, more diffuse surface gel layer. In summary, at low O_2_, the surface layer thickness is maintained, while the gradient and surface monomer conversion increase with time; at high O_2_, the surface layer thickness grows in time, while the gradient and concentration at the gel surface are maintained.

#### 3.4.4 Model Predictions

To more generally understand the transition between the surface gel layer growth profiles, we predict monomer conversion and the corresponding profile of the surface gel layer after 15 min of polymerization for a wide range of O_2_ concentrations (0.004 – 46.5 mol %) using our reaction–diffusion model (Figure S16). At very low O_2_ concentrations below 0.1 mol % (∼ 1,000 ppm), increasing the amount of O_2_ also decreases the conversion at the surface of the gel, i.e., gives rise to a gel whose surface polymer concentration is lower (Figure S17a). In this regime, consistent with the surface gel layer profile of our low O_2_ (0.01 mol %) case described above, increasing the level of O_2_ also increases the thickness of the surface gel layer (Figure S17b) though its gradient stays relatively constant (Figure S17c). Only very small amounts of O_2_ (above 0.1 mol %) cause the surface of the reacting media to remain unpolymerized (Figure S17d), which results in the gel’s surface polymer concentration being fixed at the gel point (about 5% monomer conversion). Further increasing the level of O_2_ above 0.1 mol % causes the surface gel layer thickness to grow, which in turn results in a shallower surface gel layer gradient (Figure S17c). These features are consistent with the surface gel layer profile of our high O_2_ (20 mol %) case described above. Excessively high O_2_ also impacts bulk conversion (Figure S17e), which will increase the mesh size in the gel bulk but may negatively affect the gel’s strength. We expect the predicted trends to hold for other gel compositions (monomer, cross-linker, initiator, and catalyst concentrations) and polymerization media heights (*H*) as controlled by the dimensionless groups governing this reaction–diffusion model (*Da*_M_, *Da*_O_2__, and α), though the cutoff in the O_2_ concentration above which the surface is at the gel point will be composition- and *H*-dependent. The interplay between these variables and their impact on the gradient structure at the surface of the gel are challenging to verify experimentally and represent a rich area for future study.

Moving beyond the analysis of the gelation process for the two oxygen conditions studied in this work, the results of the model provide practical principles for using oxygen- and time-dependent control of gelation reactions to control the formation of surface layers during hydrogel preparation, thereby enabling the design of superlubricity. The experiments and the model reveal that superlubricity is achieved under high oxygen polymerization conditions through the formation of a surface layer that is both thick and diffuse. These features could be enhanced by performing reactions in an oxygen-enriched atmosphere at the expense of the polymer concentration in the bulk. In general, the model could be used to predict or design preswollen surface gel layer profiles for a wide range of monomer and oxygen compositions, reaction times, and polymerization media heights. Furthermore, it is interesting to consider how time variations in the atmospheric oxygen concentration could be used to sculpt the desired shape of the surface gel layer conversion profile for a particular gel composition and geometry, thereby providing added control in the design of the structure and properties of the surface layer. Because such control can be difficult to achieve in practice, we leave a detailed study of this possibility using the model and experiments for future work.

## 4 Conclusions

Free-radical polymerization of hydrogels is an uncontrolled, random process that frustrates predictions of the precise microstructure at the surface and within the bulk. Here, we proposed that oxygen inhibition of the acrylamide/*N*,*N*′-methylenebis(acrylamide) polymerization leads to superlubricity at the hydrogel surface, with the friction coefficient decreasing 2 orders of magnitude from μ = 0.021 ± 0.006 at 0.01% O_2_ to μ = 0.002 ± 0.001 at 20 mol % O_2_. To explain these results, we developed a parameter-free reaction–diffusion model using kinetic parameters for the exact chemistry and composition of the system. We found that polymerizing under high oxygen conditions (20 mol % O_2_) increased the thickness of the surface gel layer but decreased the severity of the monomer conversion gradient between the surface and the bulk. This was indicated by a lower monomer conversion at the gel surface during the reaction, even though the gels cast at 0.01 and 20 mol % O_2_ had similar conversions in the bulk (corresponding to similar moduli). Prior to swelling the gels, we predicted that the gel at 0.01 mol % O_2_ had a surface gel layer thickness of 11 μm with a monomer conversion gradient of 4%/μm while the gel at 20 mol % O_2_ had a surface gel layer thickness of 51 μm with a 1%/μm gradient, indicating that the surface gel layer at 20 mol % O_2_ was approximately 5 times thicker and 4 times less concentrated at the surface prior to swelling. Simulations suggest that the surface layer thickness, polymer concentration gradient, and elastic modulus at the surface can be tuned to achieve the lowest friction coefficient at a given monomer/cross-linker concentration, using a combination of the O_2_ concentration and the interplay between reaction time and initiator concentration. We leave a comprehensive study of the complex interplay between these variables, their effects on friction and modulus, and their fine-tuning for specific applications to future work. Overall, this work established that a thicker surface gel layer with lower polymer concentration led to superlubricity in the polyacrylamide system under oxygen inhibition and portends the use of more sophisticated time-dependent control of reaction conditions to sculpt the precise structure—and therefore properties—of the surface gel layer.

## Supporting Information Available

The Supporting Information is available free of charge at https://pubs.acs.org/doi/10.1021/acsami.3c04636. The data that support the findings of this study are openly available in Dryad at https://doi.org/10.5061/dryad.sbcc2frcv.

Hydrogel polymerization data, contact mechanics analysis, experimental uncertainties, and tribological and mechanical properties (PDF)
