# Supplementary material for: Designing Superlubricious Hydrogels from Spontaneous Peroxidation Gradients
Source: ACS Appl Mater Interfaces. 2023 Aug 31;15(36):43075–86. doi: 10.1021/acsami.3c04636 (PMC10510045; doi:10.1021/acsami.3c04636)
Supplement: Supplementary file 1 — am3c04636_si_001.pdf [file am3c04636_si_001.pdf]

Supporting Information

Designing Superlubricious Hydrogels from Spontaneous Peroxidation Gradients

Allison L. Chau[ab+], Chelsea E.R. Edwards[bc+], Matthew E. Helgeson[bc], and Angela A. Pitenis[ab]*

[a]Materials Department, University of California, Santa Barbara, Santa Barbara, CA, 93106, United States

[b]Materials Research Laboratory, University of California, Santa Barbara, Santa Barbara, CA, 93106, United States

[c]Department of Chemical Engineering, University of California, Santa Barbara, Santa Barbara, CA, 93106, United States

[+]co-first authors

Corresponding Author:

[*]Angela A. Pitenis

apitenis@ucsb.edu

1 Comparison of Literature Data

Table S1. Comparison of the thickness of the unpolymerized oxygen inhibited layer for a variety of polymer thin film and hydrogel systems.

Acrylate System	Oxygen Inhibited Layer Thickness (µm)*	Characterization Technique	Ref.
Urethane-acrylate	1	Positron annihilation spectroscopy, X-ray photoelectron spectroscopy, microthermal analysis, nanoindentation	1
Urethane acrylate difunctional resin	4 – 34	Confocal Raman spectroscopy	2
Poly(ethylene glycol) diacrylate	4 – 35	Modeling (kinetics)	3
1,6-hexanediol diacrylate	5 – 50	Modeling (kinetics, heat transfer, mass transfer)	4
Dimethacrylate	10 – 20	Confocal Raman spectroscopy	5
Polyethylene glycol diacrylate hydrogel (40 wt.%)	20	Reaction-diffusion finite element model and experiments	6

* dependent on photoinitiator concentration, initiation rate, film thickness, oxygen concentration

2 Hydrogel Synthesis

Table S2. Volume and mass of each component in the pre-polymerized hydrogel solution, along with the corresponding wt.% and mol.%.

Chemical	Stock Solution		Mass (mg)	Mole (mmol)	Concentration	
	Concentration	Volume (µL)			wt.%	mol.%
Acrylamide (AAm)	30 wt.%	2917	875	12.3	17.5	5.15
N,N'-methylenebisacrylamide (MBAm)	2 wt.%	1750	35	0.2	0.7	0.10
DI water	---	183	4077	226	81.5	94.7
N,N,N',N'-tetramethylethylenediamine (TEMED)	10 vol.% (7.9 wt.%)	75	6.0	0.05	0.12	0.02
Ammonium persulfate (APS)	10 wt.%	75	7.5	0.03	0.15	0.01

Table S3. Synthesis conditions for the 17.5 wt.% polyacrylamide hydrogels.

Target O_2 ppm	Actual O_2 ppm (mol%)	Relative Humidity	Temperature (°C)
100 ppm (0.01 mol% O_2)	30 – 500 ppm (0.003 – 0.05 mol%)	14 – 22%	19 – 20.5
200,000 ppm (20 mol% O_2)	Ambient air (20.95 mol%)	42 – 64%	19 – 20.5

3 Hertzian Contact Mechanics

3.1 Minimizing Fitting Error

The lsqcurvefit Matlab function was used to fit the approach curves for both the microindentation and nanoindentation measurements. This function uses the squared 2-norm of the residual (**Eqn. S1**), and minimizes this value to find the desired variable, *x*.

$$\sum (f(x,x_{data}) - y_{data})^2 \tag{S1}$$

In this case, the desired variable x is the reduced elastic modulus, E^*, x_{data} are the indentation depths d, y_{data} are the normal forces F_n, and the function is $F_\text{n} = \frac{4}{3}E^* R^{1/2} d^{3/2}$.

3.2 Microindentation Hertz Fits

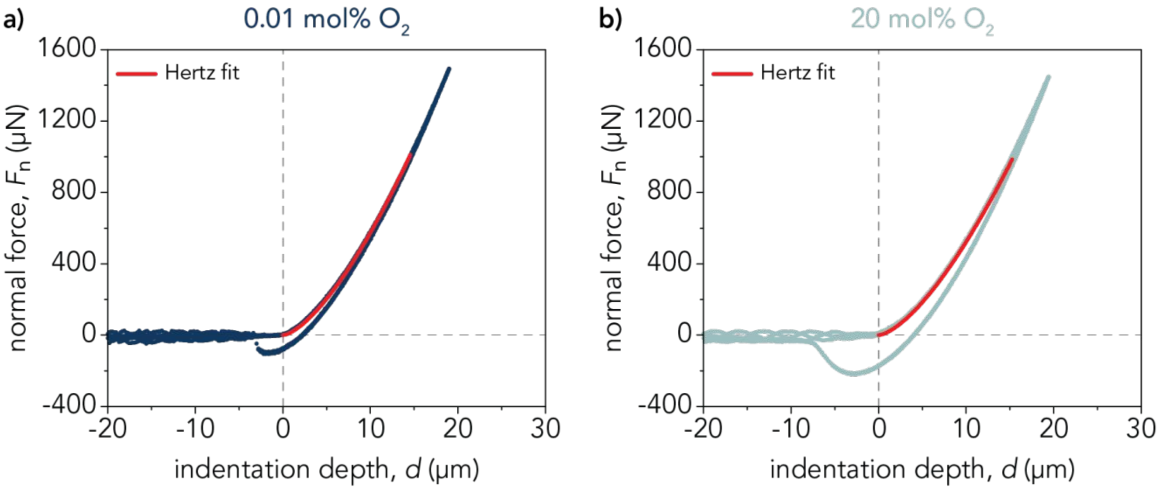

Figure S1 Representative microindentation approach curves with the Hertz contact mechanics model overlayed in red fitting from the point of contact to F_n = 1 mN for the gels cast at **(a)** 0.01 mol% O_2 and **(b)** 22 mol% O_2.

3.3 Small Angle Approximation

The small angle approximation for the trigonometric function cos is as follows:

$$\cos(\theta) = 1 - \frac{\theta^2}{2} \tag{S2}$$

The indentation depth into the sample can be predicted via geometry (**Figure S2**).

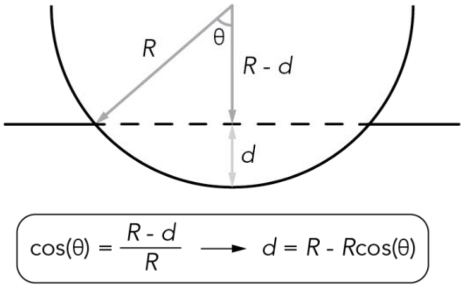

Figure S2 Schematic of probe with radius of curvature, *R*, indenting a sample with indentation depth, *d*.

Eqn. S2 can be substituted for cos(θ) so:

$$d = \frac{\theta^2}{2} R \tag{S3}$$

To stay within 1% error for the small angle approximation, $\theta = 38°$. With a probe radius of $R = 2.5$ µm for the nanoindentations, $d = 0.55$ µm.

4 Theoretical Noise Floor for Friction Coefficient Measurements

The noise floor, or minimum detectable friction coefficient, for the tribometer was estimated with the following equation:

$$\mu = \frac{F_f}{F_n} = \frac{K_f \cdot x}{F_n} \tag{S4}$$

where K_f is the tangential spring constant of the double-leaf cantilever, x is the minimum detectable displacement by the capacitance probes, and F_n is the applied normal force. For our experiments, $K_f = 100$ µN/µm. $F_n = 1$ mN, and the capacitance probes (Lion Precision, C5R-0.80-2.0) used to measure the displacement have a 5 nm resolution ($x = 5$ nm).

5 Contact Area and Pressure Estimations

The contact radius, a, was estimated based on Hertzian contact mechanics using the following equation:

$$a = \sqrt{Rd} \tag{S5}$$

where R is the probe radius and d is the indentation depth. The maximum contact radius was determined based on the maximum indentation depth reached at a normal force of F_n = 1 mN.

The maximum contact pressure was estimated using the following equation:

$$P = \frac{3F_n}{2\pi a^2} \tag{S6}$$

Table S4. Estimated contact radius, contact area, and maximum contact pressure based on Hertzian contact mechanics.

Hydrogel	Reduced Elastic Modulus, E^* (kPa)	Contact Radius, a (µm)	Contact Area, A (mm^2)	Maximum Contact Pressure, P (kPa)
0.01 mol% O_2	**242 ± 11** (3 gels, n = 54)	**213 ± 3** (3 gels, n = 54)	**0.142 ± 0.004** (3 gels, n = 54)	**11 ± 0.3** (3 gels, n = 54)
20 mol% O_2	**203 ± 38** (3 gels, n = 54)	**226 ± 3** (3 gels, n = 54)	**0.161 ± 0.028** (3 gels, n = 54)	**9 ± 1** (3 gels, n = 54)

6 Propagation and Inhibition Mechanisms for Acrylamide and Bisacrylamide

The vast structural dispersity arising from polyacrylamide propagation mechanisms, including MBAm crosslinker incorporation and reaction pathways, is explored in this section and summarized in **Figure S3**. All individual radicals (highlighted in teal) are assumed to have equivalent reactivity in all of these reactions in the model, in order to avoid accounting for the vast dispersity of local polyacrylamide chain radical structures. These radicals would all have an equivalent—but different—reactivity with oxygen, as denoted by the difference in k_p and k_s in **Table S5**. While **Figure S3** shows a number of possible reaction pathways after incorporation of a crosslinker into a growing oligomer, note that MBAm could also be the first monomer incorporated, and that the monomers can be incorporated in any sequence.

One of these reaction pathways is intramolecular cyclization, wherein the other end of an MBAm monomer already incorporated into the polymer polymerizes into the chain. While a ring formed between radicals and crosslinks within the same MBAm crosslinker was shown here, note that this process can occur for much larger cycles. Cycles are more likely to form at lower monomer concentration and are considered defects because they render the crosslinker elastically ineffective.

When the other end of an incorporated MBAm monomer does not experience cyclization, it is likely that growth of a second polyacrylamide chain will be initiated at that double-bond, establishing MBAm an elastically-effective crosslink. Note that the depiction in **Figure S3** shows the example of this initiation prior to the continued growth of the existing chain, but due to the short radical lifetimes it is likely that this initiation event occurs at a later time. Aside from cyclization, the incorporation of each end of MBAm into the gel matrix are decoupled.

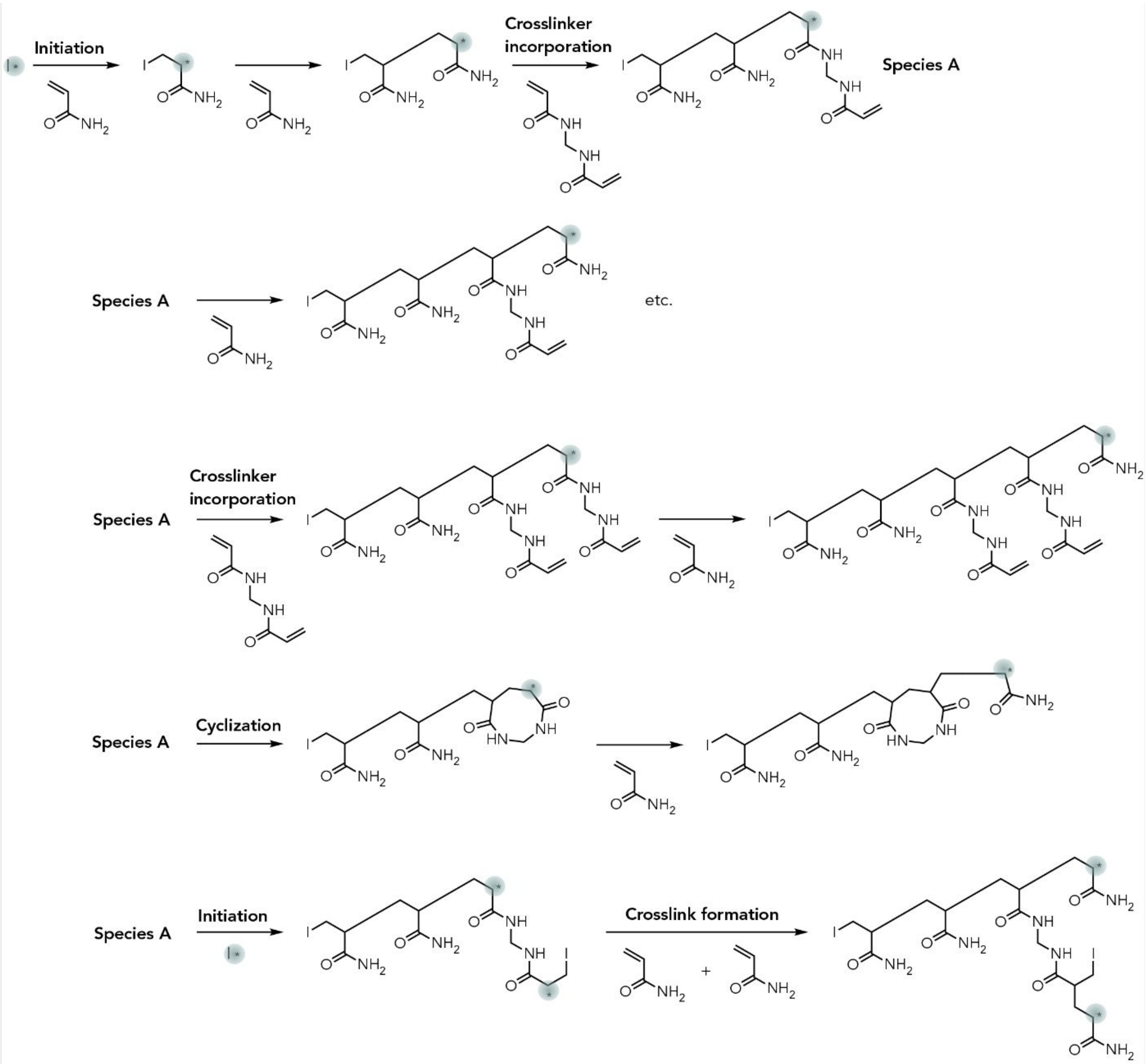

Figure S3 Structures formed during initiation and propagation of polyacrylamide; radicals are highlighted in teal for easy visualization. On initiation, the chain grows with AAm until incorporation of a crosslinker, denoted by example oligomer Species A. Once incorporated into a chain, the resulting radical structure can either react with AAm or MBAm monomer or react with the double bond at the other end of the MBAm crosslinker to form a cyclic defect. Additionally, the other double bond may come in contact with another radical initiator instead, resulting in

polyacrylamide chains continuing to grow simultaneously and independently at both ends of the crosslinker, and thus the establishment of a crosslink.

7 Reaction-Diffusion Constants and Component Concentrations

Table S5. Kinetic constants from literature and experimental reaction conditions used in our model.

Parameter	Value Used	Units	Ref.
Initiation rate constant, k_i	2.5×10^{-5}	$m^3/(mol\ s)$	7
Propagation rate constant, k_p	1	$m^3/(mol\ s)$	8
Termination rate constant, k_t	3×10^3	$m^3/(mol\ s)$	8
Inhibition rate constant, k_s	10^5	$m^3/(mol\ s)$	7
Diffusivity of O_2 in water, D_{O2}	8×10^{-10}	m^2/s	9
Depth of the reacting medium, H	0.0052	m	Calculated with 5 mL solution and 35 mm diameter mold
$[O_2]_{init,\ 0.01\%}$	0.00013088	mol/m^3	Calculated with 100 ppm O_2 at standard temperature and pressure (STP) is 0.00409 mol/m^3 (air is 40.9 mol/m^3 at STP); and Henry's constant as $H^{cc} = 0.032$.
$[O_2]_{init,\ 20\%}$	0.26176	mol/m^3	Calculated with 200,000 ppm O_2 in air is 8.18 mol/m^3; and $H^{cc} = 0.032$.
$[TEMED]_{init}$	12.90	mol/m^3	Calculated with 17.5 wt.% gel protocol
$[APS]_{init}$	6.57	mol/m^3	Calculated with 17.5 wt.% gel protocol
$[M]_{init}$	2552	mol/m^3	$[M]_{init} = [AAm]_{init} + 2[MBAm]_{init}$ Calculated with 17.5 wt.% gel protocol

Using the values listed in **Table S5**, the following constants were calculated with **Eq. S7-S11**:

$$APS_{const} = \frac{k_i H^2 [\text{TEMED}]_{init}}{D_{O_2}} \tag{S7}$$

$$TEMED_{\text{const}} = \frac{[\text{APS}]_{\text{init}}}{[\text{TEMED}]_{\text{init}}} = \tag{S8}$$

$$Da_{\text{O2}} = \frac{k_s^2 H^2 [\text{O}_2]_{\text{init}}}{2k_t D_{\text{O}_2}} = \tag{S9}$$

$$Da_{\text{M}} = \frac{k_p k_s H^2 [\text{O}_2]_{\text{init}}}{2k_t D_{\text{O}_2}} \tag{S10}$$

$$\alpha = \frac{8k_i k_t\, [\text{APS}]_{\text{init}}[\text{TEMED}]_{\text{init}}}{(k_s[\text{O}_2]_{\text{init}})^2} \tag{S11}$$

Table S6. Constants calculated for the 0.01 mol% O_2 and 20 mol% O_2 polyacrylamide hydrogels.

Constants	0.01 mol% O_2	20 mol% O_2
APS_{const}	10.9005	10.9005
$TEMED_{\text{const}}$	0.5093	0.5093
Da_{O2}	7.3729 x 10^6	1.4746 x 10^{10}
Da_{M}	73.729	147460
α	0.2967	7.4216 x 10^{-8}

We note that in simulation, the dimensionless time τ is significantly larger than the equivalent numerical value of real time *t*. When *t* = 1 sec, this corresponds to dimensionless time $\tau = \frac{t\, D_{\text{O}_2}}{H^2} =$ 2.96 x 10^{-5}. Therefore, *t* = 15 min corresponds to τ = 0.0266272.

8 Reaction-Diffusion Model Derivation

Consumption of APS and TEMED follow $\frac{dC}{dt} = -\, k_i[\text{APS}][\text{TEMED}]$, where *C* represents [APS] or [TEMED]. Introducing dimensionless APS concentration as $A = \frac{[\text{APS}]}{[\text{APS}]_{init}}$, dimensionless TEMED concentration as $T = \frac{[\text{TEMED}]}{[\text{TEMED}]_{init}}$, and dimensionless time as $\tau = \frac{t\, D_{\text{O}_2}}{H^2}$, we obtain the following dimensionless equation for APS consumption:

$$\frac{dA}{d\tau}\frac{D_{\text{O}_2}[\text{APS}]_{\text{init}}}{H^2} = -\, k_i AT[\text{APS}]_{\text{init}}[\text{TEMED}]_{\text{init}} \rightarrow \frac{dA}{d\tau} = -\frac{k_i H^2 [\text{TEMED}]_{\text{init}}}{D_{\text{O}_2}} AT \tag{S12}$$

Similarly, the dimensionless consumption of TEMED follows:

$$\frac{dT}{d\tau}\frac{D_{O_2}[\mathrm{TEMED}]_{\mathrm{init}}}{H^2} = -k_i AT[\mathrm{APS}]_{\mathrm{init}}[\mathrm{TEMED}]_{\mathrm{init}} \rightarrow \frac{dT}{d\tau} = -\frac{k_i H^2[\mathrm{APS}]_{\mathrm{init}}}{D_{O_2}}AT \quad (S13)$$

Next, nondimensionalize the reaction-diffusion equation for oxygen. Here, we introduce $\theta = \frac{[O_2]}{[O_2]_{init}}$ as dimensionless $[O_2]$ and $\eta = \frac{z}{H}$ as dimensionless depth from the surface of the reacting medium:

$$\frac{\delta[O_2]}{\delta t} = D_{O_2}\frac{\delta^2[O_2]}{\delta z^2} - k_s[R*][O_2] \rightarrow$$

$$\frac{D_{O_2}[O_2]_{\mathrm{init}}}{H^2}\frac{\delta\theta}{\delta\tau} = \frac{D_{O_2}[O_2]_{\mathrm{init}}}{H^2}\frac{\delta^2\theta}{\delta\eta^2} - k_s[R*][O_2]_{\mathrm{init}}\theta \quad (S14)$$

$$\frac{\delta\theta}{\delta\tau} = \frac{\delta^2\theta}{\delta\eta^2} - \frac{k_s[R*]H^2}{D_{O_2}}\theta \quad (S15)$$

The concentration of reactive radicals [R*] is given by:

$$\frac{-k_s[O_2] + \left((k_s[O_2])^2 + 8k_i k_t[\mathrm{APS}][\mathrm{TEMED}]\right)^{1/2}}{2k_t} \quad (S16)$$

or in terms of dimensionless variables,

$$[R*] = \frac{-k_s[O_2]_{\mathrm{init}}\theta + \left((k_s[O_2]_{\mathrm{init}})^2\theta^2 + 8k_i k_t[\mathrm{APS}]_{\mathrm{init}}[\mathrm{TEMED}]_{\mathrm{init}}AT\right)^{1/2}}{2k_t} \quad (S17)$$

Plug this expression into the partial differential equation for θ and simplify to obtain:

$$\frac{\delta\theta}{\delta\tau} = \frac{\delta^2\theta}{\delta\eta^2} - \frac{k_s{}^2H^2[O_2]_{\mathrm{init}}}{2k_t D_{O_2}}\theta\left(-\theta + \left(\theta^2 + \frac{8k_i k_t[\mathrm{APS}]_{\mathrm{init}}[\mathrm{TEMED}]_{\mathrm{init}}}{(k_s[O_2]_{\mathrm{init}})^2}AT\right)^{1/2}\right) \quad (S18)$$

By defining the constants Da_{O_2} and α (see main text), the final expression for oxygen diffusion is:

$$\frac{\delta\theta}{\delta\tau} = \frac{\delta^2\theta}{\delta\eta^2} - Da_{O_2}\theta\left(-\theta + [\theta^2 + \alpha AT]^{1/2}\right) \quad (S19)$$

Next, the reaction-diffusion equation for monomer follows $\frac{d[M]}{dt} = -k_p[R*][M]$. This expression can be nondimensionalized to find $\xi = \frac{[M]}{[M]_{init}}$, the dimensionless monomer concentration at a given depth in the reaction medium. Note that the conversion of monomer, X, which is reported in the main text and results, satisfies $X = 1 - \xi$.

$$\frac{D_{o_2}[\mathrm{M}]_{\mathrm{init}} d\xi}{H^2 \quad d\tau} = -k_{\mathrm{p}}[\mathrm{R}*][\mathrm{M}]_{\mathrm{init}}\xi \rightarrow \frac{d\xi}{d\tau} = \frac{-k_{\mathrm{p}}H^2}{D_{\mathrm{O}_2}}[\mathrm{R}*]\xi \qquad \text{(S20)}$$

Plug in [R*] to obtain:

$$\frac{d\xi}{d\tau} = \frac{-k_{\mathrm{p}}k_{\mathrm{s}}H^2[\mathrm{O}_2]_{\mathrm{init}}}{2k_{\mathrm{t}}D_{\mathrm{O}_2}}\xi\left(-\theta + \left(\theta^2 + 8\frac{k_{\mathrm{i}}k_{\mathrm{t}}}{k_{\mathrm{s}}^2}\frac{[\mathrm{APS}]_{\mathrm{init}}[\mathrm{TEMED}]_{\mathrm{init}}}{[\mathrm{O}_2]_{\mathrm{init}}^2}AT\right)^{1/2}\right) \qquad \text{(S21)}$$

Using the same definition of α and defining a new Damköhler number, $Da_{\mathrm{M}} = \frac{k_{\mathrm{p}}k_{\mathrm{s}}H^2[\mathrm{O}_2]_{\mathrm{init}}}{2k_{\mathrm{t}}D_{\mathrm{O}_2}}$, the final differential equation for monomer conversion is obtained:

$$\frac{d\xi}{d\tau} = -Da_{\mathrm{M}}\xi\left(-\theta + \left(\theta^2 + \alpha AT\right)^{1/2}\right) \qquad \text{(S22)}$$

9 Literature Comparison

Table S7. Comparison of the elastic modulus, friction coefficient, and estimations of the surface gel layer thickness of polyacrylamide hydrogels cast with different monomer (acrylamide, AAm) and crosslinker (*N,N*'-methylenebisacrylamide, MBAm) concentrations and polymerization conditions to our data (last row). The listed friction coefficient values were determined by digitizing the data from literature at the listed testing parameters (sliding speed, *v*, and estimated contact pressure, *P*).[10–20] Unless otherwise stated, the tribological testing parameters are as follows: *v* = 0.5 mm/s and *P* ≈ 6-11 kPa. Gemini indicates a self-mated gel-on-gel configuration during tribological experiments (e.g., the probe material was composed of the same hydrogel material). *Testing parameters are as follows: *v* = 100 µm/s and *P* ≈ 1 kPa ** Poisson's ratio of 0.5 was used to estimate the elastic modulus for our data (17.5 wt.% polyacrylamide hydrogels).

concentrations (wt%)		mold material (O_2 mol)	elastic modulus (E) (kPa)			friction coefficient (μ)	surface gel layer		Ref.
AAm	MBAm		micro-rheology	AFM	micro tribometer		thickness (μm)	characterization technique	
2.25		**glass** (20%)	0.12						[10]
2.5		**glass** (20%)	0.16						
2.75		**glass** (20%)	0.21						
5	0.2	**PS** (20%)			7.3		21	contact mechanics	[11]
7.5	0.03				2.4		44	contact mechanics	
7.5	0.3				34		17	contact mechanics	
7.5	0.5				40		13	contact mechanics	
15	0.6				170		13	contact mechanics	
7.5	0.3	**PS** (20%)			31-33	**Steel***: μ = 0.026 **Gemini***: μ ≈ 0.01	13-29	microindentations	[12]
7.5	0.3	**glass** (<0.01%)		11	29	**Gemini:** μ ≈ 0.05	crosslinked surface structure expected		[13]
		**PDMS** (<0.01%)		≈ 1	27	**Gemini:** μ ≈ 0.009	50	microindentations, FTIR	
7.5	0.3	**glass** (20%)		≈ 9		**Gemini**: μ ≈ 0.09	crosslinked surface structure expected		[14]
		**PS** (20%)		≈ 0.4		**Gemini**: μ ≈ 0.04	"brushy" surface structure expected		
		**PE** (20%)		≈ 0.7		**Gemini**: μ ≈ 0.04	"brushy" surface structure expected		
		**PTFE** (20%)		≈ 0.4		**Gemini**: μ ≈ 0.02	"brushy" surface structure expected		
7.5	0.39	**PS** (20%)		1.5	≈ 20 - 30	**Steel probe**: μ ≈ 0.02	"brushy" surface not characterized but observed		[15]
9.6	0.4	**glass** (20%)		30		**Gemini**: μ ≈ 0.12	crosslinked surface structure expected		[16]
		**PS** (20%)		0.3		**Gemini**: μ ≈ 0.008	10-20	nanoindentations	
9.6	0.4	**PE** (0%)		21		**Gemini**: μ ≈ 0.12	crosslinked surface structure expected		[17]
		**PE** (0.2%)		7		**Gemini**: μ ≈ 0.07	surface layer getting softer		
		**PE** (0.4%)		3		**Gemini**: μ ≈ 0.02	surface layer getting softer		
		**PE** (1%)		< 1		**Gemini**: μ ≈ 0.004	> 5	nanoindentations	
		**PE** (7.2%)		< 1		**Gemini**: μ ≈ 0.002	surface layer getting softer		
		**PE** (22%)		< 1		**Gemini**: μ ≈ 0.006	> 15	nanoindentations	

concentrations (wt%)		mold material (O_2 mol)	elastic modulus (E) (kPa)			friction coefficient (μ)	surface gel layer		Ref.
**AAm**	**MBAm**		**micro-rheology**	**AFM**	**micro tribometer**		**thickness (μm)**	**characterization technique**	
10	0.4	**glass** (20%)		29		**Gemini**: μ ≈ 0.05	crosslinked surface structure expected		[18]
		**PS** (20%)		< 0.1		**Gemini**: μ ≈ 0.02	10-20	nanoindentations	
10	0.4	**PS** (20%)		0.03			12	poroelastic contact mechanics model	[19]
3.75	0.15	**PS** (20%)				**Gemini**: μ ≈ 0.005			[20]
7.5	0.3					**Gemini**: μ ≈ 0.009			
10	0.4					**Gemini**: μ ≈ 0.01			
12.5	0.5					**Gemini**: μ ≈ 0.02			
17.5	0.7					**Gemini**: μ ≈ 0.037			
17.5	0.7	**free** (0.01%)		193**	182**	**Glass:** μ ≈ 0.02	11	reaction-kinetics model	
		**free** (20%)		0.8**	152**	**Glass**: μ ≈ 0.002	51	reaction-kinetics model	

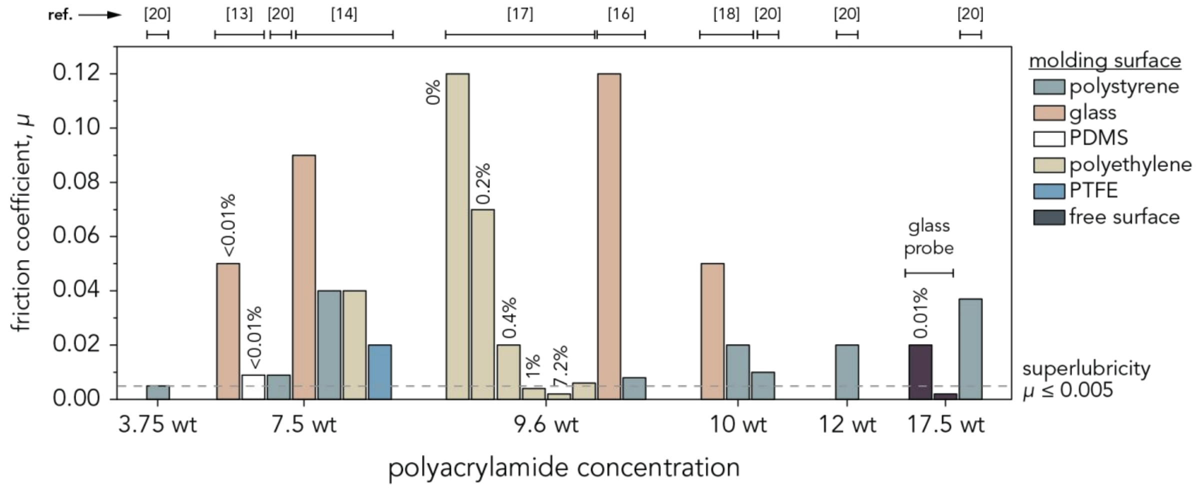

Figure S4 Comparison of friction coefficients from literature of polyacrylamide hydrogels with varying monomer and crosslinker concentrations molded against different materials (polystyrene, glass, PDMS, polyethylene, PTFE, and air-liquid interface ("free")). All samples are cast at 20 mol% O_2 unless labeled otherwise. Sliding experiments were conducted with a self-mated gel-on-gel configuration unless otherwise stated. Tribological testing parameters are as follows: v = 0.5 mm/s and $P \approx$ 6-11 kPa.

10 Friction Coefficient

10.1 Minimum Film Thickness Calculations

The Sommerfeld number, S, is a dimensionless parameter that is proportional to the viscosity of the lubricating fluid, the sliding velocity, v, and the applied normal force, F_n, as $S \,\alpha \frac{\eta v}{F_n}$.[21] The Stribeck curve plots the friction coefficient as a function of the Sommerfeld number for two sliding interfaces and partitions it into four main lubrication regimes: boundary, mixed, elastohydrodynamic lubrication (EHL), and hydrodynamic lubrication. Boundary lubrication occurs when the two sliding interfaces are in direct contact with each other whereas EHL and hydrodynamic lubrication occurs when the fluid film is thick enough to separate the two sliding interfaces. Due to this lubricating fluid layer, the friction coefficients measured within this regime are lower than those in boundary lubrication. The lubrication mode is highly dependent on applied force and sliding speed, with boundary lubrication typically occurring at lower sliding speeds and higher applied forces. Using soft-elastohydrodynamic lubrication theory developed by Hamrock and Dowson,[22] the minimum fluid film thickness, h_{min}, for soft-EHL lubrication was estimated between 33 – 36 nm using **Eqn. S23**.

$$h_{min} = 2.8R^{0.77}(\eta_o v)^{0.65}E'^{\,-0.44}F_n^{-0.21} \tag{S23}$$

where R is the probe radius of curvature, $\eta_{\mathrm{o}}$is the viscosity of the fluid, v is the sliding velocity, F_{n} is the applied normal load, and $E' = 2E^*$ where E^* is the reduced elastic modulus of the sample. The minimum fluid film thickness was calculated by using the viscosity of water (η_{o} = 8.9 x 10^{-4} Pa · s), v = 0.5 mm/s, F_{n} = 1 mN, and E^* = 200 and 240 kPa (the minimum and maximum measured E^* for the polyacrylamide hydrogels). If h_{min} was significantly greater than the surface roughness of the hydrogel, then the measured superlubricity would be more likely due to a thick fluid film layer rather than the hydrogel surface. Since the hydrogels were cast in open air, it is difficult to estimate the surface roughness. However, using the scaling relationship $\mu \sim \xi^{-1}$ developed by Urueña for self-mated gel-on-gel contacts[20] and following a Monte Carlo analysis outlined by Pitenis *et al.*,[23] the estimated mesh sizes at the surfaces of the hydrogels at 0.01 mol% and 20 mol% O_2 are ξ = 2.2 ± 0.4 nm and ξ = 31.8 ± 14.9 nm, respectively. Since h_{min} is on the same order of magnitude as the estimated mesh size of the 20 mol% O_2 gel, it is likely that there is still contact between the gel surface and probe, indicating that the measured superlubricity is not solely due to a fluid film layer.

At an applied normal force of 1 mN, the maximum pressure during sliding ranged between 9 – 11 kPa, depending on E* of the polyacrylamide hydrogel. Since $E*$ scales with osmotic pressure and the applied contact pressures are much lower than $E*$, it is unlikely that fluid flow or draining occurred, suggesting that the friction coefficients measured are not due to fluid flow.[24]

The track length was chosen so the distance was at least 4 times the contact area diameter to ensure that the probe moved out of its initial contact area zone when sliding. The minimum track lengths necessary for an applied normal force of 1 mN for hydrogels with reduced elastic moduli of 200 kPa and 240 kPa are 1.8 mm and 1.7 mm, respectively. Hertzian contact mechanics was used to estimate the contact area diameter, d (**Eqn. S24**).

$$d = 2\left(\frac{3F_n R}{4E^*}\right)^{1/3} \tag{S24}$$

10.2 Friction Coefficient Over Time

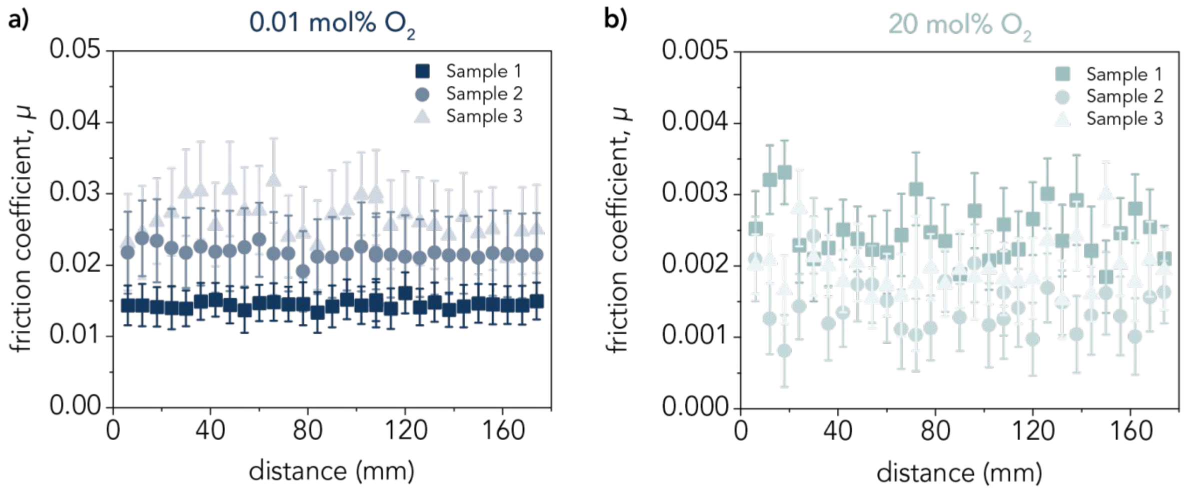

Figure S5 Friction coefficient as a function of sliding distance over 30 cycles for the **(a)** 0.01 mol% O_2 and **(b)** 20 mol% O_2. The friction does not significantly change over time. Note the difference in scale between the two plots.

11 Effects of Load and Velocity on Mechanical Properties

11.1 Friction Coefficient as a Function of Load

Previous measurements of 17.5 wt.% polyacrylamide hydrogels cast against polystyrene in ambient conditions (20 mol% O_2) and tested in self-mated contact have reported friction coefficients of $\mu = 0.037$ at an applied normal force of $F_n = 2$ mN and sliding speed of $v = 0.5$ mm/s (**Table S7**).[20] For self-mated gel-on-gel sliding configurations, friction coefficients generally decrease with increasing applied normal force since the contact radius does not scale with normal load.[25] For the 17.5 wt.% hydrogels herein, the friction coefficient slightly increased from $\mu = 0.021 \pm 0.006$ to $\mu = 0.026 \pm 0.003$ at $F_n = 2$ mN.

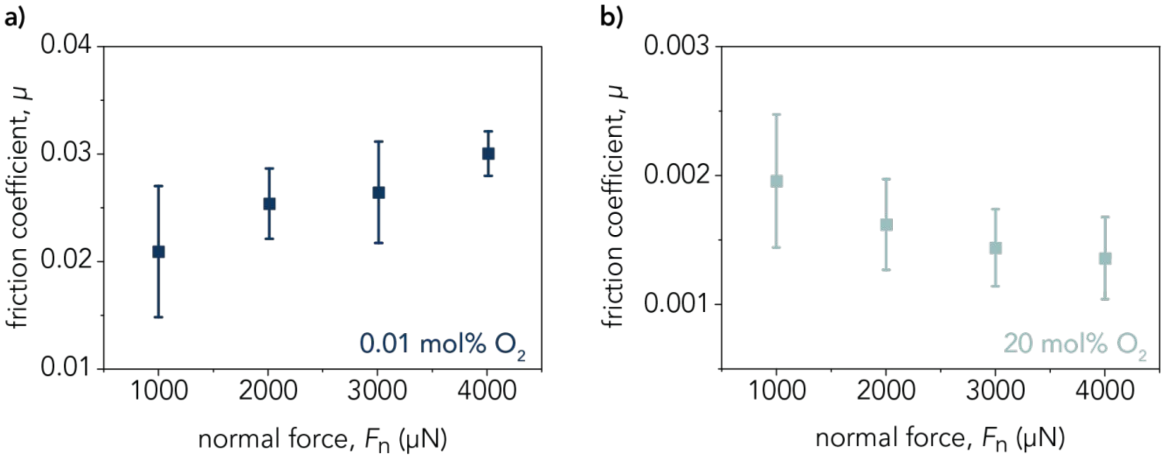

Figure S6 Friction coefficient as a function of applied normal force, F_n, for the 17.5 wt.% polyacrylamide hydrogels at 0.01 mol% O_2 and 20 mol% O_2. **(a)** Friction coefficient slightly increased with increasing normal force at 0.01 mol% O_2. **(b)** Friction coefficient slightly decreased with increasing normal force at 20 mol% O_2.

11.2 Friction Coefficient as a Function of Sliding Velocity

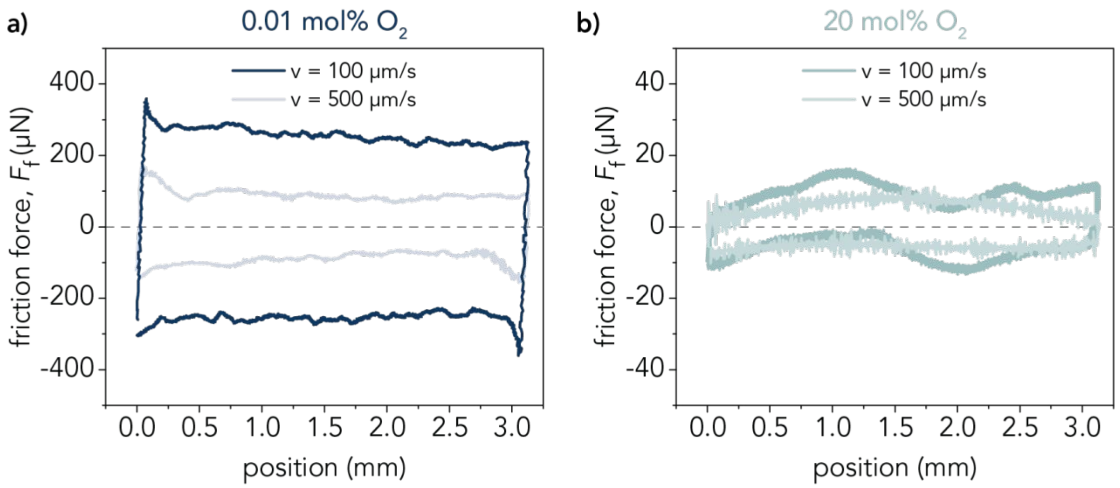

Figure S7 Friction force loops comparing the effects of sliding velocity for the 17.5 wt.% polyacrylamide hydrogels at **(a)** 0.01 mol% O_2 and **(b)** 20 mol% O_2. In both cases, the friction force decreased with increasing sliding velocity, which is the opposite of what is typically observed for hydrogels. This may indicate poroelastic effects that requires further investigation.

11.3 Elastic Modulus as a Function of Indentation Velocity

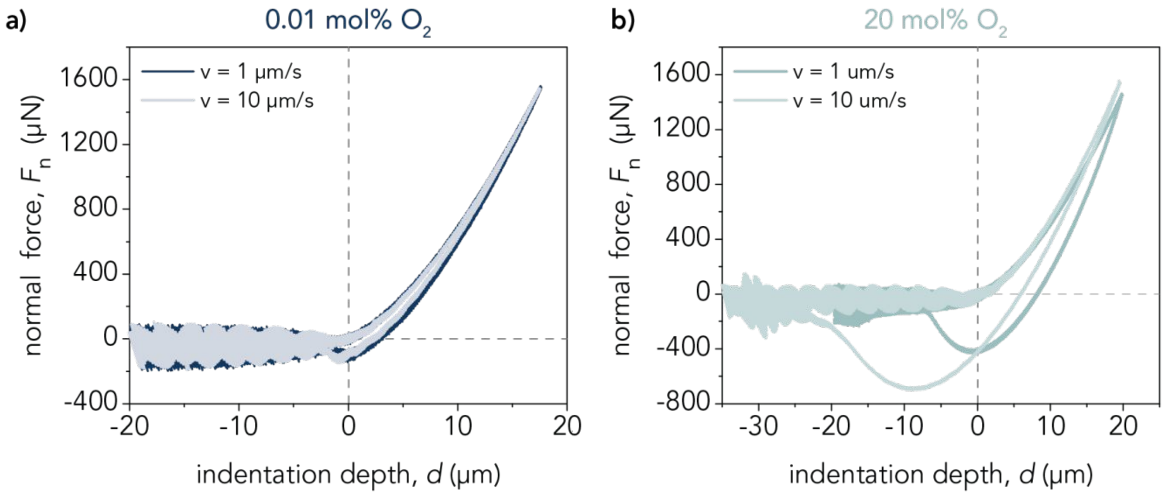

Figure S8 Representative microindentation curves for the 17.5 wt.% polyacrylamide hydrogels comparing indentation velocities. **(a)** For the 0.01 mol% O_2 hydrogels, the elastic modulus was not affected by indentation velocity. **(b)** For the 20 mol% O_2 hydrogels, there was a slight decrease (≈ 18%) in elastic modulus with decreasing indentation velocity.

12 Atomic Force Microscopy

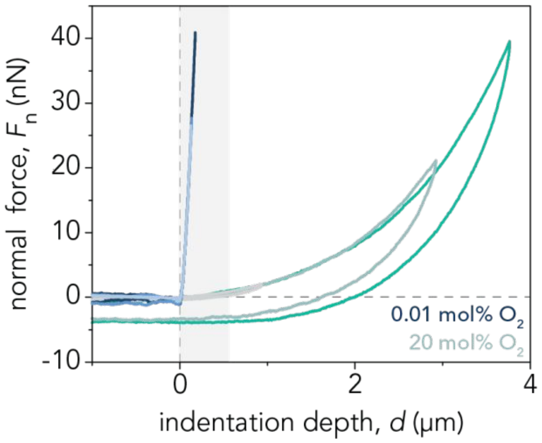

Figure S9 Full nanoindentation curves for the 17.5 wt.% polyacrylamide hydrogels at 0.01% O_2 (dark blue) and 20% O_2 (light blue) for varying applied normal forces (F_n = 2, 20, 25, 30, 40 nN) at an indentation velocity of 2 μm/s. The region of analysis (the first 0.55 μm) is indicated by the gray region.

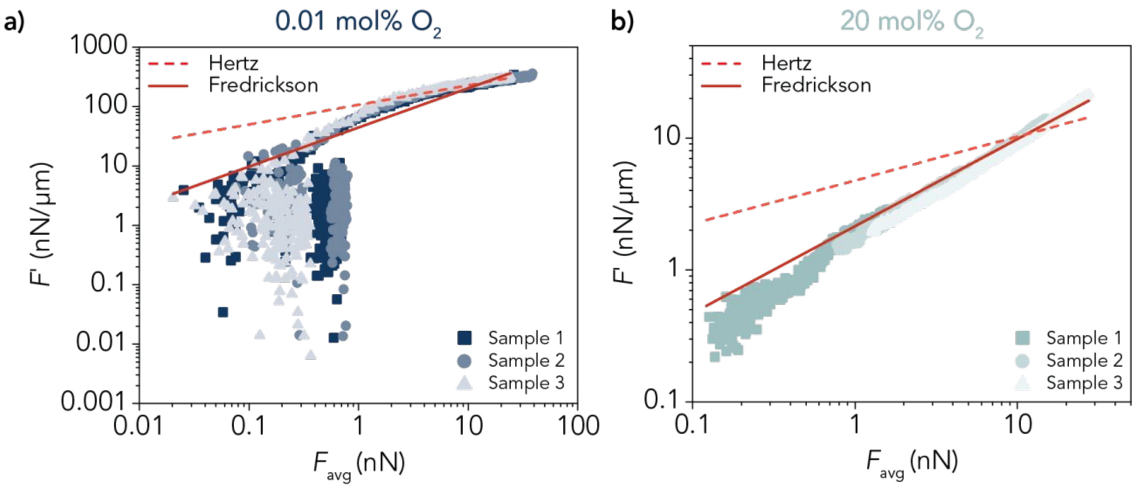

Figure S10 Representative log-log plots of the derivative (dF_n/dd) of the nanoindentation approach curves as a function of force following the methods outlined by Garcia *et al*.[26] The dashed red line represents the Hertzian contact model while the solid red line represents the Fredrickson high-penetration brush model.[11,27,28] **(a)** At 0.01 mol% O_2, the Hertz contact model fits the data better. **(b)** At 20 mol% O_2, the Fredrickson high-penetration brush contact model fits the data better.

13 Extracting Surface Layer Thickness and Gradient

A series of studies by Baselga *et al*. in the late 1980s sought to determine the process of network formation for AAm/MBAm gelation with APS and TEMED initiator under various conditions.[29] One such study determined that a sample with initial $[AAm]_{init}$ = 1.28 mol/L and $[MBAm]_{init}$ = 0.074 mol/L reached the gel point at 6% monomer conversion based on digitization of graphical data provided in that paper.[29] In a separate study, Baselga *et al*. found that doubling the concentration of monomer and crosslinker, while halving the weight percent of crosslinker in the initial feedstock—similar to our experimental system with $[AAm]_{init}$ = 2.46 mol/L and $[MBAm]_{init}$ = 0.05 mol/L—approximately halved the conversion (by weight) at the gel point.[30] As a safe estimate, we assumed gelation occurred at 5% monomer conversion (rather than 3%) in this work. Thus, according to our model, the entirety of this surface gel layer reached gelation.

The first point where the monomer conversion exceeds 5% is taken to be the upper surface of the gel. To minimize error of fit while capturing surface shape, a depth of gel is chosen for fitting such that the surface gel layer represents about 10%. In other words, for the 0.01 mol% O_2 case, the top 100 µm of gel is fit to extract surface gel layer thickness and gradient, whereas the top 300 µm is fit for the 20 mol% O_2 case. Fitting is conducted on this cropped data at each time point using the MATLAB shape language modeling (SLM) engine. A degree 1 fit was conducted using 3 knots and free interior knots; *i.e*., two line segments were fit to the data. The thickness of the surface gel layer was taken to be the depth corresponding to the intersection of these two line segments. The slope of the gradient layer was taken to be the slope of the line at the surface of the gel.

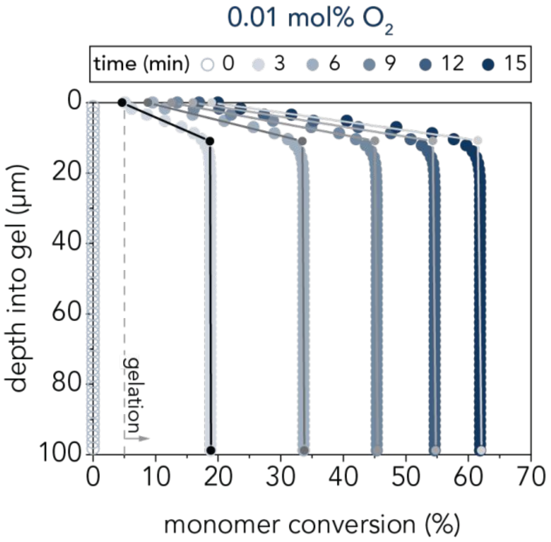

Figure S11 Fits of monomer conversion for the top 100 μm of gel for the hydrogels polymerized at 0.01 mol% O_2. The gray lines show the exact line segment fit of monomer conversion, with the intersection of the line segments denoting the transition from surface gel layer to the bulk gel. Fits are shown at various reaction times. SLM algorithm fits well at all time points.

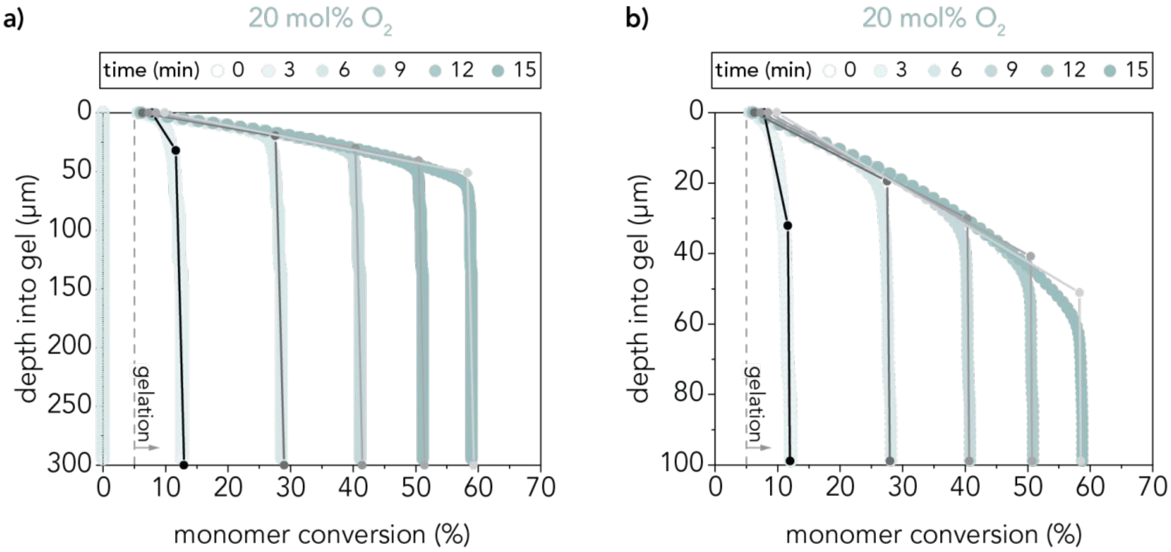

Figure S12 Fits of monomer conversion for the top **(a)** 300 μm and **(b)** 100 μm of gel for the hydrogels polymerized at 20 mol% O_2. The gray lines show the exact line segment fit of monomer conversion, with the intersection of the line segments denoting the transition from surface gel layer to the bulk gel. Fits are shown at various reaction times. The SLM algorithm fits well at all time points except 3 minutes, where the root mean square error (RMSE) value is above the cutoff; clearly the surface and bulk layers are not yet distinct at 3 minutes.

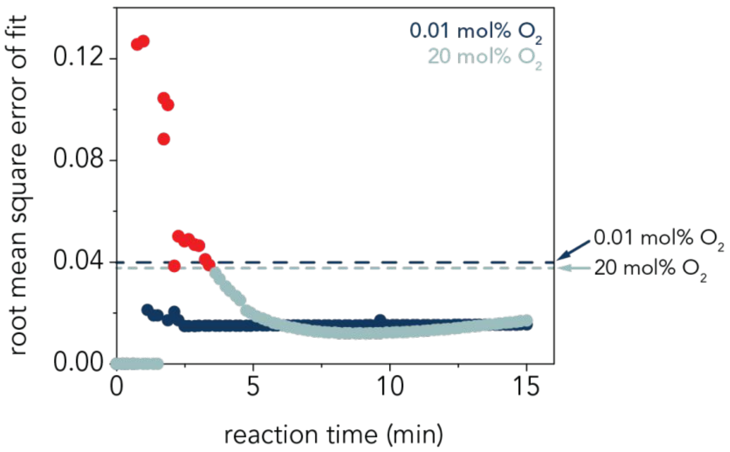

Figure S13 Root mean square error (RMSE) of the SLM fit for 0.01 mol% O_2 (dark blue) and 20 mol% O_2 (light blue). Horizontal lines indicate the RMSE cutoff value, one standard deviation above the average of the non-zero RMSE values at each O_2 concentration. Above the cutoff, the fits do not accurately reflect the shape of the surface layer, corresponding to an ill-defined distinction between the surface gel layer and bulk gel as the gel forms at early times. The red dots represent the reaction times in which the corresponding thickness and gradient of the surface gel layer were excluded from the main text.

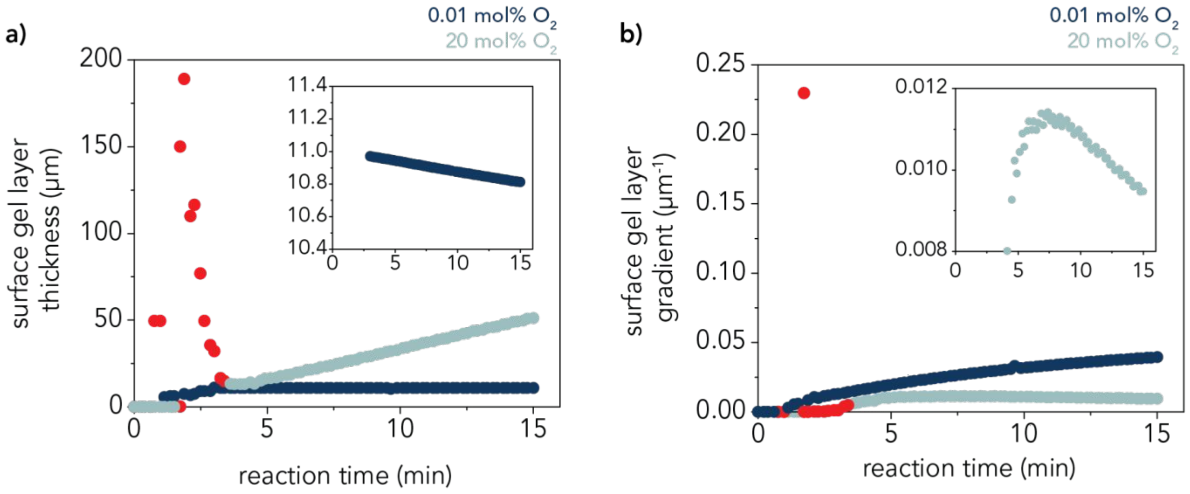

Figure S14 (a) Surface gel layer thickness and **(b)** surface gel layer thickness gradient as a function of reaction time with insets highlighting the decrease in surface gel layer thickness and gradient with increasing time. The red dots represent the reaction times in which the RMSE of the SLM fit was greater than the cutoff value and excluded from the main text.

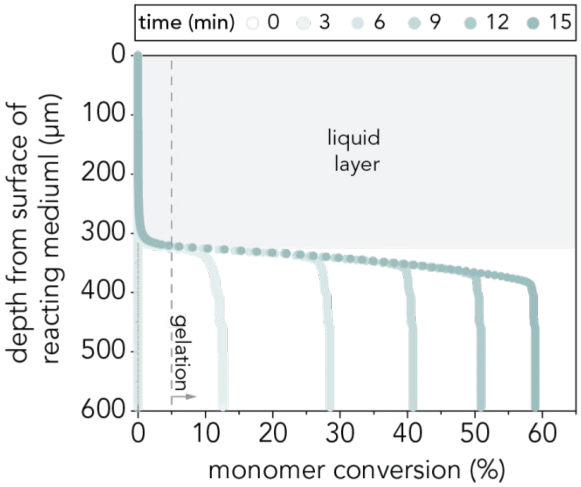

Figure S15 Unlike the 0.01 mol% O_2 case, in the 20 mol% O_2 polymerization shown here, oxygen inhibits the polymerization at the surface of the reaction so that even after 15 minutes, no significant monomer conversion or gelation occurs until about 300 μm into the reacting medium, resulting in a thinner gel.

14 Model Results for Varying Oxygen Concentration

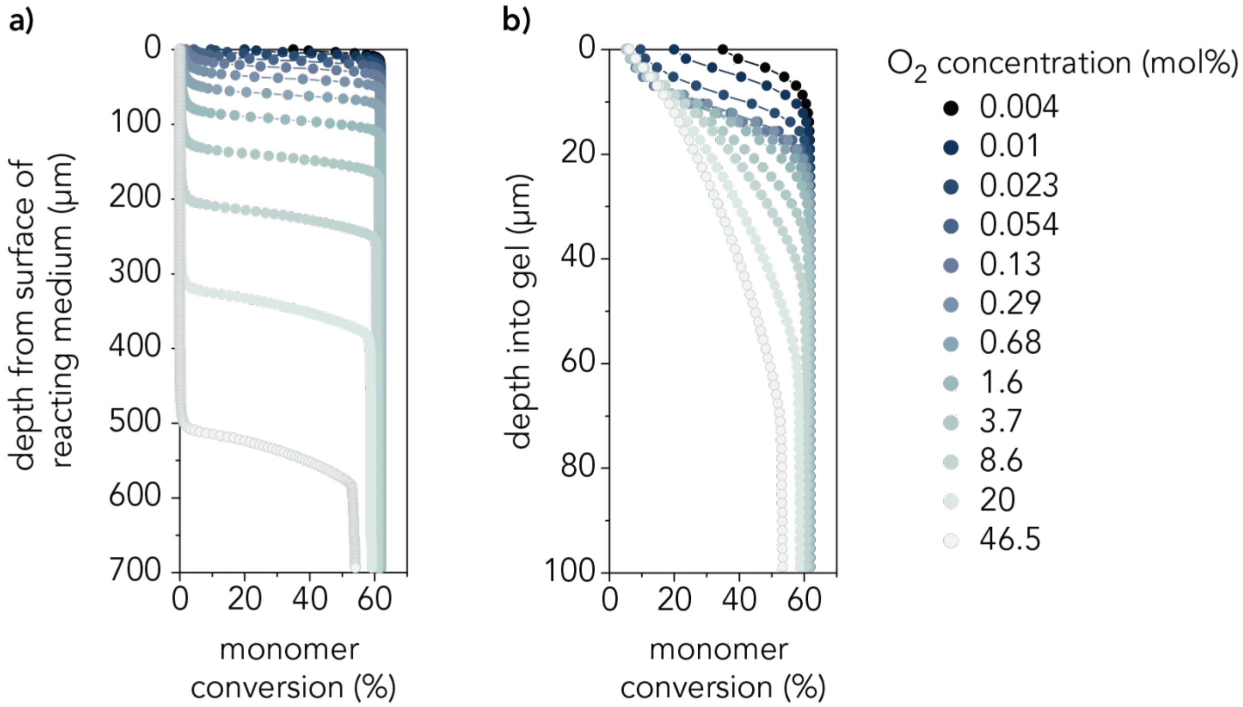

Figure S16 Monomer conversion fraction profiles after 15 minutes of polymerization at various oxygen concentrations (0.004 – 46.5 mol%), with all other parameters fixed (monomer, crosslinker, and initiator concentrations; reaction medium depth *H*; and kinetic constants). Profiles

are shown **(a)** as a function of depth from the top surface of the reacting medium and **(b)** as a function of depth into the gel prior to swellings by removing any ungelled liquid layer (< 5% conversion) at the upper surface of the reacting medium.

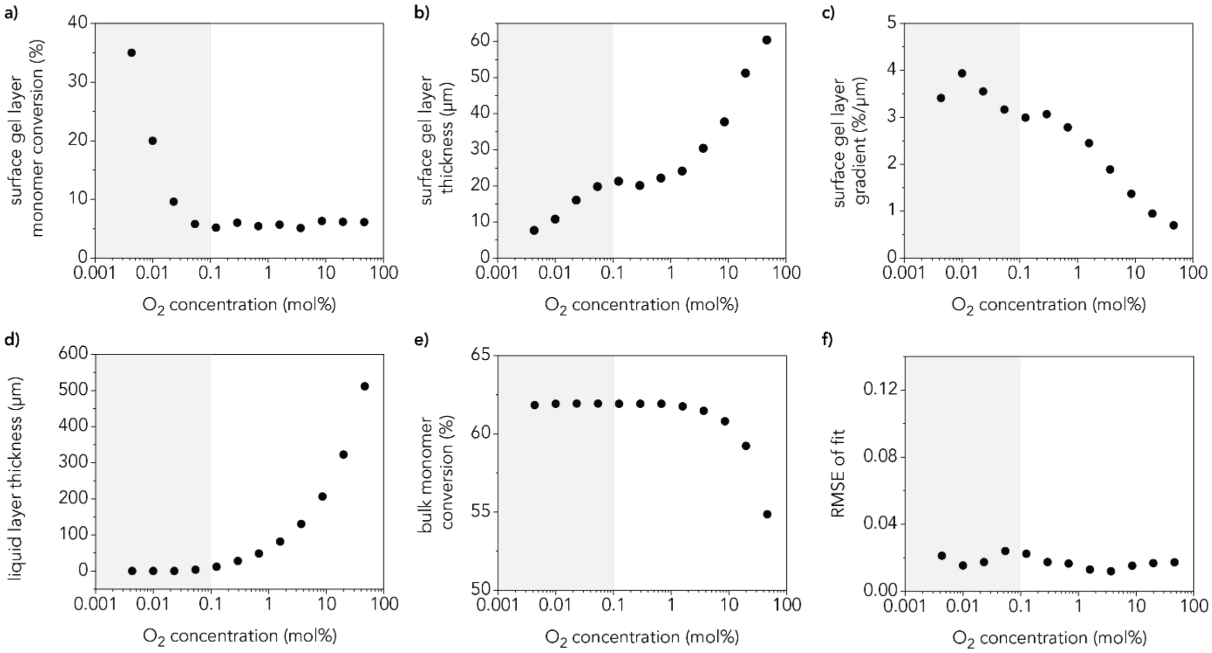

Figure S17 The following are plotted at varying oxygen concentrations corresponding to the various profiles plotted in **Figure S16**. **(a)** Conversion of monomer at the surface of the gel. **(b)** Thickness of the surface gel layer from SLM fit of the profiles. **(c)** Monomer conversion gradient in the surface gel layer, also extracted from the SLM fit of the profiles. **(d)** Thickness of the unpolymerized liquid layer at the upper surface of the reacting medium. **(e)** Monomer conversion in the gel bulk, as measured by the conversion deep into the gel, which is affected by oxygen diffusion only at early times during polymerization. **(f)** Root mean square error (RMSE) of fit corresponding to **(b)** and **(c)**. Note that the RMSE cutoff for the 0.01 mol% and 20 mol% O_2 data reported in the text is around 0.04, so all values reported here fit well.

15 Data Compilation

Table S8. Compilation of the mechanics, friction coefficient, and surface gel layer thickness for the 17.5 wt.% polyacrylamide hydrogels at 0.01 mol% and 20 mol% O_2.

	**Reduced elastic modulus, E^* (kPa), tribometer**	**Reduced elastic modulus, E^* (kPa), AFM**	**Friction coefficient, μ**	**Surface gel layer thickness before swelling, t**
**0.01 mol% O_2**	**242 ± 11**<br>(3 gels, n = 54)	**257 ± 48**<br>(3 gels, n = 15)	**0.021 ± 0.006**<br>(n = 3 gels)	10.8 µm with<br>4 %/µm gradient
**20 mol% O_2**	**203 ± 38**<br>(3 gels, n = 54)	**1.1 ± 0.2**<br>(2 gels, n = 10)	**0.002 ± 0.001**<br>(n = 3 gels)	51.2 µm with<br>1 %/µm gradient

16 References

(1) Cao, H.; Currie, E.; Tilley, M.; Jean, Y. C. Oxygen Inhibition Effect on Surface Properties of UV-Curable Acrylate Coatings. In *ACS Symposium Series*; 2003; pp 152–164. https://doi.org/10.1021/bk-2003-0847.ch013.

(2) Courtecuisse, F.; Karasu, F.; Allonas, X.; Croutxé-Barghorn, C.; van der Ven, L. Confocal Raman Microscopy Study of Several Factors Known to Influence the Oxygen Inhibition of Acrylate Photopolymerization under LED. *Prog Org Coat* **2016**, *92*, 1–7. https://doi.org/10.1016/j.porgcoat.2015.11.020.

(3) Dendukuri, D.; Panda, P.; Haghgooie, R.; Kim, J. M.; Hatton, T. A.; Doyle, P. S. Modeling of Oxygen-Inhibited Free Radical Photopolymerization in a PDMS Microfluidic Device. *Macromolecules* **2008**, *41*, 8547–8556. https://doi.org/10.1021/ma801219w.

(4) O'Brien, A. K.; Bowman, C. N. Modeling the Effect of Oxygen on Photopolymerization Kinetics. *Macromol Theory Simul* **2006**, *15* (2), 176–182. https://doi.org/10.1002/mats.200500056.

(5) Pierrel, J.; Ibrahim, A.; Croutxé-Barghorn, C.; Allonas, X. Effect of the Oxygen Affected Layer in Multilayered Photopolymers. *Polym Chem* **2017**, *8* (31), 4596–4602. https://doi.org/10.1039/c7py00974g.

(6) Krutkramelis, K.; Xia, B.; Oakey, J. Monodisperse Polyethylene Glycol Diacrylate Hydrogel Microsphere Formation by Oxygen-Controlled Photopolymerization in a Microfluidic Device. *Lab Chip* **2016**, *16* (8), 1457–1465. https://doi.org/10.1039/C6LC00254D.

(7) Kulichikhin, S. G.; Malkin, A. Y.; Polushkina, O. M.; Kulichikhin, V. G. Rheokinetics of Free-Radical Polymerization of Acrylamide in an Aqueous Solution. *Polym Eng Sci* **1997**, *37* (8), 1331–1338. https://doi.org/10.1002/pen.11779.

(8) Decker, C.; Jenkins, A. D. Kinetic Approach of O2 Inhibition in Ultraviolet and Laser-Induced Polymerizations. *Macromolecules* **1985**, *18* (6), 1241–1244. https://doi.org/10.1021/ma00148a034.

(9) Hepworth, S. J.; Leach, M. O.; Doran, S. J. Dynamics of Polymerization in Polyacrylamide Gel (PAG) Dosimeters: (II) Modelling Oxygen Diffusion. *Phys. Med. Biol.* **1999**, *44*, 1875–1884.

(10) Pedro, D. I.; Nguyen, D. T.; Trachsel, L.; Rosa, J. G.; Chu, B.; Eikenberry, S.; Sumerlin, B. S.; Sawyer, W. G. Superficial Modulus, Water-Content, and Mesh-Size at Hydrogel Surfaces. *Tribol Lett* **2021**, *69* (4), 1–9. https://doi.org/10.1007/s11249-021-01538-3.

(11) Johnson, C. L.; Dunn, A. C. Composition Controls Soft Hydrogel Surface Layer Dimensions and Contact Mechanics. *Biointerphases* **2022**, *17* (6), 061002. https://doi.org/10.1116/6.0002047.

(12) Johnson, C. L.; Dunn, A. C. Tribological Characterization of Gradient-Density Polyacrylamide Hydrogel Surfaces. *Exp Mech* **2021**, *61* (5), 829–842. https://doi.org/10.1007/s11340-021-00704-x.

(13) Gombert, Y.; Simič, R.; Roncoroni, F.; Dübner, M.; Geue, T.; Spencer, N. D. Structuring Hydrogel Surfaces for Tribology. *Adv Mater Interfaces* **2019**, *6* (22), 1901320. https://doi.org/10.1002/admi.201901320.

(14) Meier, Y. A.; Zhang, K.; Spencer, N. D.; Simic, R. Linking Friction and Surface Properties of Hydrogels Molded Against Materials of Different Surface Energies. *Langmuir* **2019**, *35* (48), 15805–15812. https://doi.org/10.1021/acs.langmuir.9b01636.

(15) Bonyadi, S. Z.; Atten, M.; Dunn, A. C. Self-Regenerating Compliance and Lubrication of Polyacrylamide Hydrogels. *Soft Matter* **2019**, *15* (43), 8728–8740. https://doi.org/10.1039/c9sm01607d.

(16) Simič, R.; Mandal, J.; Zhang, K.; Spencer, N. D. Oxygen Inhibition of Free-Radical Polymerization Is the Dominant Mechanism behind the "Mold Effect" on Hydrogels. *Soft Matter* **2021**, *17* (26), 6394–6403. https://doi.org/10.1039/D1SM00395J.

(17) Simič, R.; Spencer, N. D. Controlling the Friction of Gels by Regulating Interfacial Oxygen During Polymerization. *Tribol Lett* **2021**, *69* (3), 86. https://doi.org/10.1007/s11249-021-01459-1.

(18) Simič, R.; Yetkin, M.; Zhang, K.; Spencer, N. D. Importance of Hydration and Surface Structure for Friction of Acrylamide Hydrogels. *Tribol Lett* **2020**, *68*, 64. https://doi.org/doi.org/10.1007/s11249-020-01304-x.

(19) Chau, A. L.; Cavanaugh, M. K.; Chen, Y. T.; Pitenis, A. A. A Simple Contact Mechanics Model for Highly Strained Aqueous Surface Gels. *Exp Mech* **2021**, *61* (4), 699–703. https://doi.org/10.1007/s11340-021-00699-5.

(20) Urueña, J. M.; Pitenis, A. A.; Nixon, R. M.; Schulze, K. D.; Angelini, T. E.; Sawyer, W. G. Mesh Size Control of Polymer Fluctuation Lubrication in Gemini Hydrogels. *Biotribology* **2015**, *1–2*, 24–29. https://doi.org/10.1016/j.biotri.2015.03.001.

(21) Shaw, M. C.; Nussdorfer Jr., T. J. *An Analysis of the Full-Floating Journal Bearing*; 1947. https://doi.org/10.1142/9789814343060_0080.

(22) Hamrock, B. J.; Dowson, D. Elastohydrodynamic Lubrication of Elliptical Contacts for Materials of Low Elastic Modulus I — Fully Flooded Conjunction. *Transactions of the ASME* **1978**, *100*, 236–244.

(23) Pitenis, A. A.; Urueña, J. M.; Cooper, A. C.; Angelini, T. E.; Sawyer, W. G. Superlubricity in Gemini Hydrogels. *J Tribol* **2016**, *138* (4), 21–23. https://doi.org/10.1115/1.4032890.

(24) de Gennes, P. G. *Scaling Concepts in Polymer Physics*; Cornell University Press: Ithaca, 1979.

(25) Urueña, J. M.; McGhee, E. O.; Angelini, T. E.; Dowson, D.; Sawyer, W. G.; Pitenis, A. A. Normal Load Scaling of Friction in Gemini Hydrogels. *Biotribology* **2018**, *13*, 30–35. https://doi.org/10.1016/j.biotri.2018.01.002.

(26) Garcia, M.; Schulze, K. D.; O'Bryan, C. S.; Bhattacharjee, T.; Sawyer, W. G.; Angelini, T. E. Eliminating the Surface Location from Soft Matter Contact Mechanics Measurements. *Tribology - Materials, Surfaces & Interfaces* **2017**, *11* (4), 187–192. https://doi.org/10.1080/17515831.2017.1397908.

(27) Klein, J.; Kamiyama, Y.; Yoshizawa, H.; Israelachvili, J. N.; Fredrickson, G. H.; Pincus, P.; Fetters, L. J. Lubrication Forces between Surfaces Bearing Polymer Brushes. *Macromolecules* **1993**, *26* (21), 5552–5560. https://doi.org/10.1021/ma00073a004.

(28) Fredrickson, G. H.; Ajdari, A.; Leibler, L.; Carton, J. P. Surface Modes and Deformation Energy of a Molten Polymer Brush. *Macromolecules* **1992**, *25* (11), 2882–2889. https://doi.org/10.1021/ma00037a015.

(29) Baselga, J.; Llorente, M. A.; Hernández-Fuentes, I.; Piérola, I. F. Polyacrylamide Gels. Process of Network Formation. *Eur Polym J* **1989**, *25* (5), 477–480. https://doi.org/10.1016/0014-3057(89)90189-4.

(30) Baselga, J.; Llorente, M. A.; Hernández-Fuentes, I.; Piérola, I. F. Network Defects in Polyacrylamide Gels. *Eur Polym J* **1989**, *25* (5), 471–475. https://doi.org/10.1016/0014-3057(89)90188-2.
